# Response to IL-6 trans- and IL-6 classic signalling is determined by the ratio of the IL-6 receptor α to gp130 expression: fusing experimental insights and dynamic modelling

**DOI:** 10.1186/s12964-019-0356-0

**Published:** 2019-05-17

**Authors:** Heike Reeh, Nadine Rudolph, Ulrike Billing, Henrike Christen, Stefan Streif, Eric Bullinger, Monica Schliemann-Bullinger, Rolf Findeisen, Fred Schaper, Heinrich J. Huber, Anna Dittrich

**Affiliations:** 1 Department of Systems Biology, Institute of Biology, Faculty of Natural Sciences, Otto-von-Guericke University Magdeburg, Universitätsplatz 2, 39106 Magdeburg, Germany; 2 Department of Systems Theory and Automatic Control, Institute for Automation Engineering, Faculty of Electrical Engineering and Information Technology, Otto-von-Guericke University Magdeburg, Universitätsplatz 2, 39106 Magdeburg, Germany; 3 Automatic Control and System Dynamics Laboratory, Institute of Automation, Chemnitz University of Technology, Reichenhainer Straße 70, 09107 Chemnitz, Germany; 4 Comuptational Biology, Discovery Research, Boehringer Ingelheim Pharma, Birkendorfer Straße 65, 88400 Biberach, Germany

**Keywords:** Inflammation, Signal transduction, Interleukin-6, IL-6, Classic signalling, Trans-signalling, Jak/STAT signalling, IL-6 receptor α, gp130, Systems biology, Computational dynamic modelling, Set-based modelling and analysis

## Abstract

**Background:**

Interleukin-6 is a pleiotropic cytokine with high clinical relevance and an important mediator of cellular communication, orchestrating both pro- and anti-inflammatory processes. Interleukin-6-induced signalling is initiated by binding of IL-6 to the IL-6 receptor α and subsequent binding to the signal transducing receptor subunit gp130. This active receptor complex initiates signalling through the Janus kinase/signal transducer and activator of transcription pathway. Of note, IL-6 receptor α exists in a soluble and a transmembrane form. Binding of IL-6 to membrane-bound IL-6 receptor α induces anti-inflammatory classic signalling, whereas binding of IL-6 to soluble IL-6 receptor α induces pro-inflammatory trans-signalling. Trans-signalling has been described to be markedly stronger than classic signalling. Understanding the molecular mechanisms that drive differences between trans- and classic signalling is important for the design of trans-signalling-specific therapies. These differences will be addressed here using a combination of dynamic mathematical modelling and molecular biology.

**Methods:**

We apply an iterative systems biology approach using set-based modelling and validation approaches combined with quantitative biochemical and cell biological analyses.

**Results:**

The combination of experimental analyses and dynamic modelling allows to relate the observed differences between IL-6-induced trans- and classic signalling to cell-type specific differences in the expression and ratios of the individual subunits of the IL-6 receptor complex. Canonical intracellular Jak/STAT signalling is indifferent in IL-6-induced trans- and classic signalling.

**Conclusion:**

This study contributes to the understanding of molecular mechanisms of IL-6 signal transduction and underlines the power of combined dynamical modelling, model-based validation and biological experiments. The opposing pro- and anti-inflammatory responses initiated by IL-6 trans- and classic signalling depend solely on the expression ratios of the subunits of the entire receptor complex. By pointing out the importance of the receptor expression ratio for the strength of IL-6 signalling this study lays a foundation for future precision medicine approaches that aim to selectively block pro-inflammatory trans-signalling. Furthermore, the derived models can be used for future therapy design.

**Graphical abstract:**

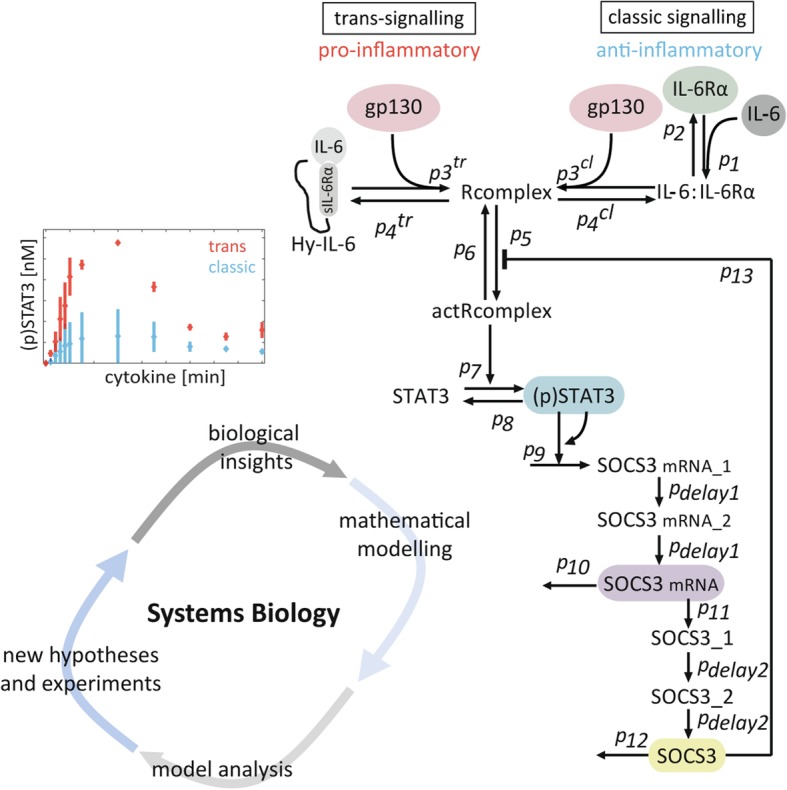

**Electronic supplementary material:**

The online version of this article (10.1186/s12964-019-0356-0) contains supplementary material, which is available to authorized users.

## Plain English summary

Cytokines - also known as tissue hormones - are soluble mediators of cell-to-cell communication within the body. Interleukin-6 is a prominent cytokine that transmits information about inflammatory processes. Interestingly, IL-6 can both boost and reduce inflammation. To transmit information IL-6 binds to cells via a specific receptor complex involving two proteins called IL-6 receptor α and gp130. IL-6 receptor α exists in two forms. One form is integrated in the cell membrane of the target cell. The other form occurs soluble in the blood serum. The receptor gp130 is located in the cell membrane. Anti-inflammatory messages are transmitted by binding of IL-6 to IL-6 receptor α in the cell membrane. This process is called classic signalling. Pro-inflammatory messages are transmitted by binding of IL-6 to soluble IL-6 receptor α. This process is called trans-signalling. Until now, the molecular mechanisms underlying the differences in response to trans- and classic signalling are unknown. We use systems biology, which is an interconnected combination of molecular biology and computational modelling and model-based analyses, to show that the ratio of membrane-bound IL-6 receptor α to gp130 on the surface of a cell determines how a cell senses trans- and classic signalling. With these results we pave the way for specific pharmaceutical inhibition of pro-inflammatory trans-signalling in inflammatory diseases.

## Background

The pleiotropic cytokine Interleukin-6 (IL-6) is a central mediator of cellular communication and is involved in the regulation of inflammatory responses as well as in the coordination of developmental, neuronal, and metabolic processes [1]. In hepatocytes, IL-6 is a major mediator of the acute-phase response [2]. Due to this crucial role in inflammation, dysregulated IL-6-induced signalling is associated with the development of severe immunological and proliferative diseases such as rheumatoid arthritis (RA), inflammatory bowel disease (IBD), and colon cancer [3, 4].

The IL-6 receptor complex consists of the IL-6-specific α receptor IL-6Rα (gp80, CD126) and the signal transducing subunit glycoprotein 130 (gp130, CD130). IL-6 first binds to IL-6Rα with low affinity. The IL-6:IL-6Rα complex subsequently builds a high affinity complex with gp130. The formation of this entire receptor complex induces subsequent activation of intracellular signalling pathways, leading to IL-6-dependent gene expression and IL-6-dependent cellular responses such as proliferation, migration, or metabolic changes [5].

Both subunits of the IL-6 receptor complex naturally occur in transmembrane and soluble forms, with the latter being generated by proteolytic cleavage or alternative splicing [6–9]. Soluble IL-6Rα (sIL-6Rα) is an agonist of IL-6-induced signalling and allows cells that do not express transmembrane IL-6Rα to respond to IL-6. Signalling via membrane-bound IL-6Rα is denoted as classic signalling, while signalling via soluble IL-6Rα is denoted as trans-signalling. Classic signalling induces regenerative and protective responses, whereas trans-signalling induces rather pro-inflammatory activities [10].

While all cells in the body express gp130, the expression of IL-6Rα is restricted to hepatocytes and leucocyte subtypes [5, 11]. Therefore, classic signalling is restrained to a small subset of cells, whereas all somatic cells are targets of trans-signalling. In contrast to sIL-6Rα, the soluble form of gp130 (sgp130) inhibits IL-6-induced signalling by sequestering IL-6:sIL-6Rα complexes [12, 13].

In order to analyse trans-signalling independently of classic signalling, Hyper-IL-6 (Hy-IL-6) was developed, which is a fusion protein composed of sIL-6Rα and IL-6 being connected by a flexible protein linker [14]. In human hepatoma cells (HepG2) Hy-IL-6 induces maximal expression of acute-phase proteins at molar concentrations that are substantially lower than those required when stimulating with IL-6 [14]. In mouse models, injection of Hy-IL-6 results in a much stronger expression of acute-phase proteins than achieved with IL-6 [15]. These observations led to the assumption that IL-6 trans-signalling is a more potent activator of intracellular signalling than IL-6 classic signalling, yet the molecular basis for this hypothesis is still elusive.

Formation of the entire receptor complex by either classic or trans-signalling induces the activation of gp130-associated Janus kinases (Jak) and results in the phosphorylation of tyrosine residues within the cytoplasmic part of gp130 [16, 17]. These phosphorylated tyrosine residues serve as binding sites for the transcription factor signal transducer and activator of transcription 3 (STAT3) [18–20]. After recruitment to the receptor, STATs are phosphorylated by Jaks, and phosphorylated STAT3-dimers translocate into the nucleus to induce STAT-specific gene expression [21]. As such, STAT3 induces the expression of suppressor of cytokine signalling 3 (SOCS3), a potent and crucial negative regulator of Jak/STAT signalling that prevents excessive inflammation [22–24].

Computational modelling and analysis of signal transduction provides means for developing and testing hypotheses about complex signalling pathways. They have been employed to study the pathological deregulations of signalling pathways in clinical context [25, 26]. By now, several computational models of the Jak/STAT pathway exist [27–36], including models specifically analysing initiation [32] and negative regulation of Jak/STAT signalling [27, 29, 34, 35] as well as crosstalk of Jak/STAT signalling and MEK/ERK pathway activation [36] in classic signalling.

However, IL-6 trans- and classic signalling have not been systematically investigated up- or downstream of receptor activation to explain the differences observed. Addressing this question requires dynamic computational modelling approaches that can discriminate different hypotheses on biological processes based on given data. To test for invalid model hypotheses that are not consistent with measured quantitative biochemical and molecular data we provided and applied methods based on statistical inference and set-based modelling and analysis approaches from non-convex optimisation [37, 38]. For the first time, these methods are applied to a realistic, rather large pathophysiological problem. Using the derived models and set-based analyses, we demonstrate that the differences in signalling observed in response to IL-6-induced trans- and classic Jak/STAT signalling are not due to molecular mechanisms downstream of the receptor, which are specific for classic or trans-signalling. Instead, the observed differences originate from differences in the molar expression ratios of soluble or membrane-bound IL-6Rα to gp130.

## Methods

### Modelling of IL-6-induced trans- and classic signalling

We used a system of ODEs to dynamically model the reaction network, which were subsequently discretized. To validate different hypotheses we used dynamic time varying measurements. The considered signalling pathways are depicted in Fig. 2b of the results section. All reactions were modelled as mass action kinetics except for SOCS3 negative feedback, which was modelled by a rational function. Please refer to the Additional file 1: Text S2.1 as well as Additional file 1: Tables S1 and S2 for the dynamic equations, initial conditions and further descriptions. The following modelling assumptions were made:

1. The system is well mixed. Because of the large number of cells, we neglect stochastic effects.
2. Since no obvious difference in STAT3 activation in response to trans-signalling-induced by Hy-IL-6 or by the IL-6:sIL-6Rα complex was identified (Additional file 1: Figure S1), we did not incorporate binding of IL-6 to soluble IL-6Rα during trans-signalling in our model.
3. IL-6 and Hy-IL-6 are assumed as constant model inputs as no change in the amount of cytokine in the supernatant was observed experimentally during the considered time horizon of 90 min (data not shown).
4. The hexameric receptor complex (IL-6:IL-6Rα:gp130)_2_ formed during classic signalling and the hexameric receptor complex (Hy-IL-6:gp130)_2_ formed during trans-signalling are considered as active receptor complexes (actRcomplex) [39].
5. Formation of the active receptor complex induces activation of receptor-associated Jaks [16, 17] and subsequent phosphorylation of tyrosine residues in the cytoplasmatic region of gp130 [40, 41]. However, as Jaks are constitutively associated with gp130 [42] we do not explicitly consider soluble Jaks in our model. Rather, we assume Jaks to be represented as part of the gp130 state variable.
6. The activated receptor complexes represent activated Jaks [17]. STATs are phosphorylated by the active receptor complex [16, 18].
7. To describe nonlinear dynamics of SOCS3 mRNA transcription, we add a positive feedback to (p)STAT3-induced SOCS3 mRNA transcription [29].
8. Negative feedback inhibition via SOCS3 [24] is modelled by a rational term to allow for an inhibition of the receptor activity [31].
9. The species SOCS3 mRNA_1, SOCS3 mRNA_2, SOCS3_1 and SOCS3_2 are modelled to simulate the delay caused by SOCS3 mRNA transcription, mRNA processing and translation, respectively. To this end, we apply a linear chain, where the delays are distributed in two steps with the kinetic rates p_delay1_ and p_delay2,_ respectively [31].

The system’s equations were derived by balancing the entities $x(t)\in {\mathbb{R}}^{{\mathrm{n}}_{\mathrm{x}}}$ (here relative and absolute concentrations of proteins and mRNA). The law of mass-action was applied to describe the reaction rates. The resulting model equations are given by:

1 $$ \dot{x}(t)=f\left(x(t),u(t),p\right),\kern0.75em x(0)={x}_0, $$

where $u(t)\in {\mathbb{R}}^{{\mathrm{n}}_{\mathrm{u}}}\ \mathrm{and}\ p\in {\mathbb{R}}^{{\mathrm{n}}_{\mathrm{p}}}$ denote the time-variant model inputs (cytokine), and the time-invariant model parameters, respectively. Additionally, ${x}_0\in {\mathbb{R}}^{{\mathrm{n}}_{\mathrm{x}}}$ denoted the initial conditions for the considered state variables (see Additional file 1: Table S3 for a description) and $f:{\mathbb{R}}^{n_x}\times {\mathbb{R}}^{n_u}\times {\mathbb{R}}^{n_p}\to {\mathbb{R}}^{n_x}$ are polynomial or rational functions. The model output equations, which relate to the experimental measurements are given by:

2 $$ y(t)=h\left(y(t),x(t),u(t),p\right), $$

where $y(t)\in {\mathbb{R}}^{{\mathrm{n}}_{\mathrm{y}}}$ denote the time-variant model outputs (here measurable states, i.e. (p)STAT3, SOCS3 mRNA and SOCS3) and $h:{\mathbb{R}}^{n_y}\times {\mathbb{R}}^{n_x}\times {\mathbb{R}}^{n_u}\times {\mathbb{R}}^{n_p}\to {\mathbb{R}}^{n_y}$ are assumed as polynomial functions.

In this study we used quantitative Western blotting and qRT-PCR data to infer the unknown model parameters, which are not available from literature. Instead of mean values we used the 1-sigma confidence intervals

3 $$ {\boldsymbol{y}}_{\boldsymbol{i}}\left({\mathbf{t}}_{\mathbf{j}}\right):= \left[\underset{\_}{{\boldsymbol{y}}_{\boldsymbol{i}}}\left({\mathbf{t}}_{\mathbf{j}}\right),\overline{{\boldsymbol{y}}_{\boldsymbol{i}}}\left({\mathbf{t}}_{\mathbf{j}}\right)\right], $$

at the measurement time-points *t*_*j*_ = {0, 1, …*n*_*t*_} and *y*_*i*_(t_j_) given by the lower ($\underset{\_}{y_i}$:= mean value – STD) and upper ($\overline{y_i}$:= mean value + STD) bounds of the measurable states *y*_*i*_(*t*_*j*_).

Initial conditions for the state variables, the initial concentrations of the signalling molecules, inputs, outputs, and parameters were assumed to lie within the (possibly large) sets ${\mathcal{X}}_0,\mathcal{U},\mathcal{Y}\kern0.2em \mathrm{and}\kern0.2em \mathcal{P}$:

4 $$ {\boldsymbol{x}}_{\mathbf{0}}\in {\mathbf{\mathcal{X}}}_{\mathbf{0}},\boldsymbol{u}\in \mathbf{\mathcal{U}},\boldsymbol{y}\in \mathbf{\mathcal{Y}},\boldsymbol{p}\in \mathbf{\mathcal{P}}. $$

**Fig. 1:**
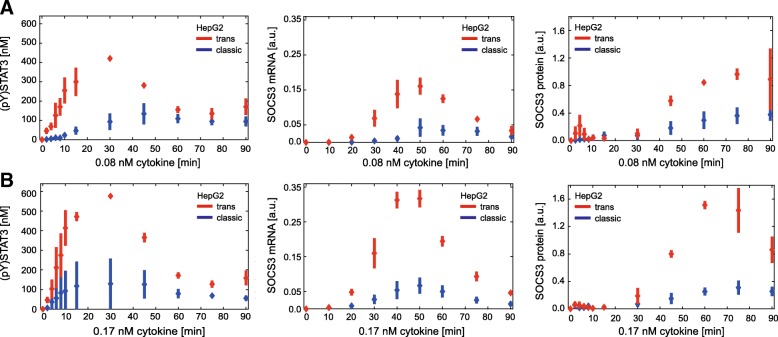
In HepG2 cells trans-signalling is stronger than classic signalling. HepG2 cells were stimulated with 0.08 nM (**a**) or 0.17 nM (**b**) IL-6 (blue) or Hy-IL-6 (red). STAT3 phosphorylation and expression of STAT3 protein, SOCS3 protein, and HSC70 protein were evaluated by Western blotting. Expression of STAT3 and HSC70 served as loading control. The expression of SOCS3 mRNA was analysed using qRT-PCR. Diamonds correspond to the mean and bars to the standard deviation of *n* = 3–4 experiments. Data normalization was performed as described in Additional file 1: Text S3

We set the initial conditions according to Additional file 1: Table S2 and the input (cytokine concentrations) according to the considered experiment. Furthermore, initial boundaries on the model parameters *p* and ranges for dissociation constants were set as given in Additional file 1: Table S3 and model outputs according to the experimental data (Figs. 1 and 3c-d in the results section) of the form in eq. (3).

### Set-based parameter estimation

**Fig. 2:**
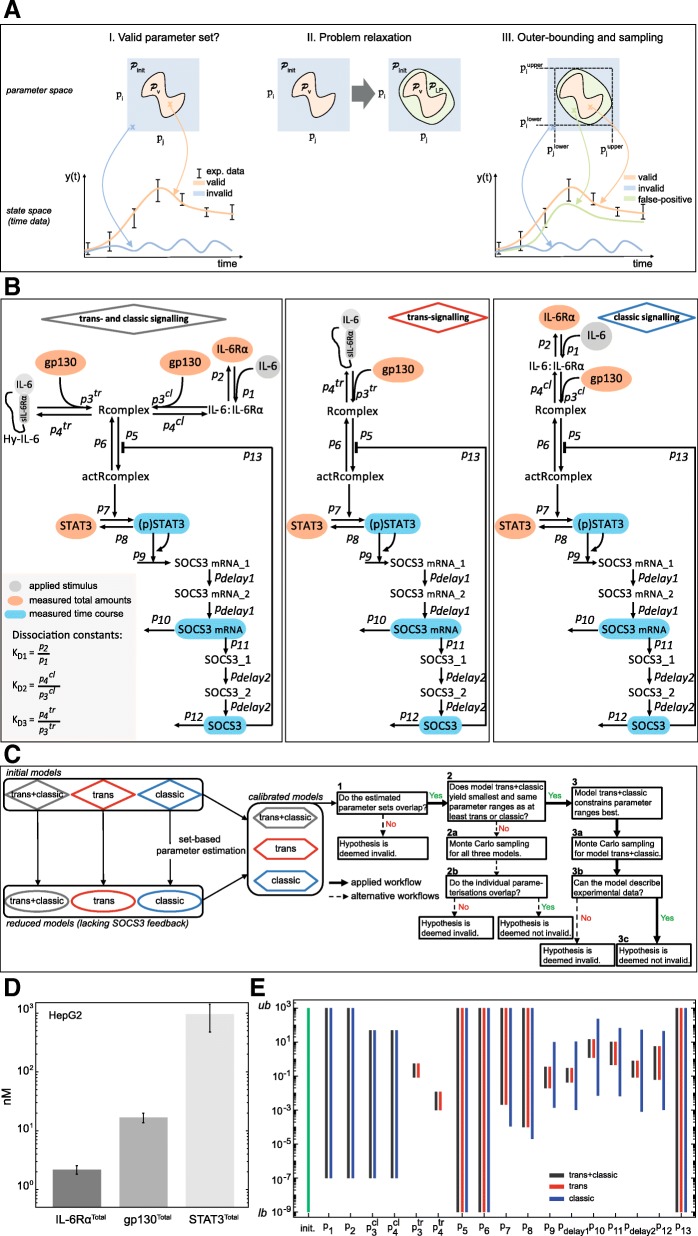
Set-based modelling, network topologies, workflow diagram and parameter estimation results. **a** I): To calculate model trajectories that describe experimental data, valid parameters (orange cross and trajectory) have to be distinguished from invalid ones (blue cross and trajectory). The orange area describes a set ${\mathcal{P}}_{\mathrm{v}}$ of valid parameters that represent data. Due to non-convexity of ${\mathcal{P}}_{\mathrm{v}}$, relaxations are performed obtaining a linear program (LP). II) Relaxations result in the set ${\mathcal{P}}_{\mathrm{LP}}$ that covers ${\mathcal{P}}_{\mathrm{v}}$, but introduce also false positive solutions (green area). III) Due to relaxations, the introduced false parameter sets give model trajectories that are inconsistent with the data (green cross and trajectory). However, it is guaranteed that the valid solution set is always included. As consequence, a model is deemed invalid, whenever ${\mathcal{P}}_{\mathrm{LP}}$ and thus, ${\mathcal{P}}_{\mathrm{v}\kern0.5em }$ are empty. We apply an outer-bounding algorithm to approximate ${\mathcal{P}}_{\mathrm{LP}\kern0.5em }$ (black dotted rectangle). **b** Initial models describing trans- and classic signalling, trans-signalling only and classic signalling only. Classic signalling is induced by binding of IL-6 to IL-6Rα. The complex associates with gp130. Trans-signalling is induced by binding of Hy-IL-6 to gp130. In both cases the active receptor complex initiates Jak/STAT signalling and SOCS3 expression. **c** Workflow for set-based parameter estimation and (non-)invalidity test. Black bold arrows depict the applied workflow, while dotted arrows show alternative workflows. **d** Expression of gp130 and IL-6Rα in HepG2 cells was quantified by flow cytometry using QIFIKIT. Mean ± STD values from *n* = 4 independent experiments are shown. Expression of STAT3 in HepG2 cells was quantified by Western blotting using recombinant calibrator proteins. Mean ± STD values from *n* = 7 independent experiments are shown. **e** Results for outer-bounding of model parameters. Initial parameter bounds (green bar) range from 10^− 9^ (lower bound, *lb*) to 10^3^ (upper bound, *ub*). Dark grey, blue and red bars depict ranges for parameters after set estimation of the models

For model analyses, we used a set-based model analysis approach, based on suitable time discretization of (1–2) as outlined in Rumschinski et al. [43]. This method allows to 1) rigorously prove invalidity of mathematical models, and thus invalidate biological hypotheses of the according model, 2) account for measurement uncertainty, and 3) exploit the fact that the model parameters are not exactly known and ranges of valid values can be determined (Fig.2a I in the results section). Technically, this set-based method checks for valid parameter sets by defining and solving a so-called mathematical feasibility problem for the dynamical model equations and constraints.

In a first step, a discrete-time approximation of our ODE models was derived, applying a first order Euler discretisation scheme. To do so, a step size of 2 min for the first 30 min of stimulation and a step size of 2.5 min for the remaining time horizon are used, i. e. between 30 and 90 min. Subsequently, the discretised model equations, experimental data and the uncertainty descriptions of the model parameters and state variables were combined into a single feasibility problem which was subsequently relaxed into a linear program (Fig. 2a II in the results part) and solved using Cplex [1]. In total, the feasibility problem describing trans- and classic signalling within one model consisted of 41 time steps, 17 unknown parameters, 10 state variables for classic signalling, 9 state variables for trans-signalling with a total size of 1335 variables. For separated analyses of trans- and classic signalling, each feasibility problem consisted of 41 time steps, 15 unknown parameters for classic signalling, 13 unknown parameters for trans-signalling with the size of the problem being 720 variables for classic signalling and 635 variables for trans-signalling. To confine the unknown parameter sets, an outer-bounding algorithm was applied giving a box-shaped outer approximation (Fig. 2 AIII in the results part). For a technical description of the relaxation steps, we refer to Additional file 1: Text S1 and references therein.

The formulation of the feasibility problems, the relaxation processes, and the algorithm for obtaining outer approximations of the parameters are implemented in the MATLAB-based toolbox ADMIT [37], which was used for model analysis in this study.

### Determining valid parameterisations using Monte Carlo sampling

Due to the relaxation approach, set-based outer approximations still possibly include false positive parameterisations that lead to model predictions being inconsistent with the data. While for an invalidation of a biological hypothesis this can be sufficient, it does not allow an in-depth inside into the signalling dynamics. To refine the results we applied Monte Carlo sampling to obtain parameter samples that can reasonable represent the experimental data. We determined 150,000 random parameter sets sampled from the estimated outer bounds of all parameters using a log2-uniform distribution. Values for IL-6Rα^Total^, gp130^Total^, and STAT3^Total^ were randomly determined within their experimentally measured uncertainty ranges, i. e. mean value ± STD (Fig. 2d in the results part). The obtained parameterisations were tested whether they can represent the experimental data using the original continuous-time models (see Additional file 1: Table S1 for equations and Fig. 4b in the results part). One hundred fifty simulations that were in line with the data and that resulted in the lowest achievable quadratic distance between simulations and corresponding data were bundled into corridors for the quantities [(p)STAT3], [SOCS3 mRNA], and [SOCS3] (Fig. 4b in the results part).

### Identifiability test using Data2Dynamics

The ODE model describing combined IL-6-induced trans- and classic signalling was implemented in the Data2Dynamics toolbox [44]. The estimated outer bounds for our initial model were set as lower and upper parameter bounds, and experimental data (Fig. 1 and 2d in the results part) were added. To calculate profile likelihoods of all parameters the function *arPLEInit* was used.

### Cultivation of cells

Ba/F3-gp130-IL-6Rα cells [45] were grown in DMEM+GlutaMax (Thermo Fisher Scientific, Waltham, MA, USA) supplemented with IL-6 (10 ng/ml) (Conaris, Kiel, Germany), FCS (10%) (#10270, Thermo Fisher Scientific), streptomycin, and penicillin (each 100 μg/ml, Thermo Fisher Scientific) at 37 °C in a water saturated atmosphere containing 5% CO_2_. Prior to stimulation, 10^6^ Ba/F3-gp130-IL-6Rα cells were washed three times with PBS and subsequently starved in 2.5 ml medium without IL-6, FCS, streptomycin, and penicillin for 2 h. Cells were treated with IL-6 (Conaris, Kiel, Germany), Hy-IL-6 (Conaris), and cycloheximide (50 μg/ml, Sigma Aldrich, St. Louis, MO, USA) as indicated in the text and figure legends.

HepG2 cells were grown in DMEM+F12 (Thermo Fisher Scientific) supplemented with FCS (10%), streptomycin and penicillin (each 100 μg/ml) at 37 °C in a water saturated atmosphere containing 5% CO_2_. HepG2 cells were retrovirally transduced with cDNA coding for human IL-6Rα as described earlier [29]. HepG2-IL-6Rα cells were grown in DMEM+F12 (Thermo Fisher Scientific) supplemented with FCS (10%), puromycin (2 μg/ml) (Carl Roth, Karlsruhe, Germany), streptomycin, and penicillin (each 100 μg/ml) at 37 °C in a water saturated atmosphere containing 5% CO_2_. For stimulation experiments 7.5 × 10^5^ HepG2 or 5 × 10^5^ HepG2-IL-6Rα cells were cultured on 6 cm dishes for 24 h. Prior to stimulation cells were starved overnight in medium without FCS, streptomycin, penicillin, and puromycin. Cells were treated with IL-6 (Conaris, Kiel, Germany), Hy-IL-6 (Conaris), and cycloheximide (50 μg/ml, Sigma Aldrich, St. Louis, MO, USA) as indicated in the text and figure legends.

### Cell viability assay

Ba/F3-gp130-IL-6Rα cells were washed three times with PBS. Subsequently, 5000 cells/well were seeded in transparent 96-well plates. Cells were incubated in the presence of IL-6 or Hy-IL-6 as indicated in the text and figure legends and if indicated with Baricitinib, Tofacitinib, Ruxolitinib (Selleckchem, Munich, Germany), or DMSO. After 48 h viability of cells was determined using Cell Titer Blue reagent (Promega, Madison, WI, USA) according to manufacturer’s instructions. Absorption at 570 nm and 600 nm was recorded with an Infinite M200 PRO reader (Tecan, Männedorf, Switzerland). Curve fitting for calculation of absolute IC50 was done with a maximum likelihood 4 parameter logistic regression implemented in MATLAB R2016b (MathWorks, Natick, MA, USA).

### Western blotting

Cells were lysed in RIPA lysis buffer (50 mM Tris-HCl pH 7.4, 150 mM NaCl, 0,5% NP-40, 15% Glycerol), supplemented with aprotinin, leupeptin and pepstatin (10 μg/ml of each), Pefabloc (0.8 μM) (Roche, Mannheim, Germany), NaF (1 mM), and Na_3_VO_4_ (1 mM). Protein concentrations of the lysates were determined using BCA assay according to manufacturer’s instructions (Thermo Fisher Scientific). Proteins were separated by SDS-Page and transferred to a polyvinylidenedifluorid membrane. Antigens were detected by incubation with specific primary antibodies (1:1000) followed by incubation with horseradish-peroxidase-coupled secondary antibodies (1:5000) (Thermo Fisher Scientific). List of primary antibodies: (p)STAT3-Y705 (#9145), STAT3 (#9139) (Cell Signaling Technology, Danvers, MA, USA); SOCS3 (#18391) (IBL, Fujioka, Japan); HSC70 (#sc-7009) (Stress Marq, Victoria, Canada). Detection was performed via ECL solution [46] using an ImageQuant LAS-Mini 4000 gel documentation system (GE Healthcare, Chalfont St Giles, UK). Quantification of bands was performed using Image Quant (GE healthcare).

Absolute protein amounts of STAT3 and (p)STAT3 per cell were analysed by quantitative immunoprecipitation as described earlier using recombinant STAT3 as calibrator (GST-STAT3 (aa 670–770), Abnova, Taipei, Taiwan) (see Additional file 1: Figure S2) [29].

Data normalization: Data from independent biological experiments were normalized as described in Additional file 1: Text S3.1.

### Flow cytometry

7.5 × 10^5^ HepG2 or HepG2-IL-6Rα cells and 10^6^ Ba/F3-gp130-IL-6Rα cells were seeded and starved as described above. After stimulation with IL-6 or Hy-IL-6 as indicated, HepG2 and HepG2-IL-6Rα cells were detached from cell culture dishes with 1 ml Accutase (Biowest, Nuaillé, France, Cat. No. L0950–100). For fixation 100 μl of the cell suspension was mixed with 100 μl paraformaldehyde and incubated for 10 min at 37 °C followed by centrifugation (230 g at 4 °C for 5 min). Cells were suspended in ice cold 90% methanol and incubated on ice for 10 min. Subsequently, cells were washed twice with cold BSA-EDTA-Buffer (2% BSA, 2 mM EDTA in PBS) and incubated with fluorophore-coupled antibodies against STAT3 (#560391) (1:50) and (p)STAT3 (#557814) (1:200) (BD Biosciences, Franklin Lakes, NJ, USA) overnight. Cells were washed two times in BSA-EDTA Buffer and applied to FACS analysis (FACS Canto II (BD Biosciences)). Data were evaluated using FlowJo (Treestar, Ashland, OR, USA).

Specificity of the fluorophore-coupled antibody against (p)STAT3 was validated in STAT3-deficient MEF cells (Additional file 1: Figure S3) and confirmed absence of unspecific binding. To independently show dose-dependent phosphorylation of STAT3 in HepG2 cells STAT3 phosphorylation in response to a wide range of Hy-IL-6 doses originally analysed was examined by using Western blotting (Additional file 1: Figure S4).

The amount of gp130 and IL-6Rα on the cell surface was analysed using QIFIKIT, a bead-based FACS assay, according to manufacturer’s instruction (Agilent, Santa Clara, CA, USA) (see Additional file 1: Figure S5).

### Quantitative RT-PCR

Total RNA was isolated using the RNeasy Kit (Qiagen, Hilden, Germany) according to manufacturer’s instructions. One microgram of RNA was reverse transcribed into cDNA with RevertAid RT Reverse Transcription Kit (Thermo Fisher Scientific), employing random hexameric primers (Thermo Fisher Scientific) according to manufacturer’s instructions. mRNA expression of SOCS3 and SDHA was analysed with primers for human SOCS3 (fw: 5′-GGA GTT CCT GGA CCA GTA CG-3′, rev: 5′-TTC TTG TGC TTG TGC CAT GT -3′) and human SDHA (fw: 5′- TGG GAA CAA GAG GGC ATC TG-3′, rev: 5′- CCA CCA CTG CAT CAA ATT CAT G-3′). PCR was performed using Maxima SYBR Green qPCR Master Mix (Thermo Fisher Scientific) according to manufacturer’s instructions. The PCR reaction was done in a final volume of 20 μl containing 2 μl cDNA. After denaturing for 10 min at 95 °C amplification was performed in 40 cycles (15 s at 95 °C, 30 s at 60 °C, 30 s at 72 °C) on a Rotorgene (Qiagen).

Data normalization: The gene of interest and the housekeeping gene were amplified in technical duplicates. Quantification of gene expression was calculated as described by Pfaffl et al. [47]. Data normalization is described in Additional file 1: Text S3.2.

## Results

### IL-6-induced trans-signalling is more potent than classic signalling in human hepatoma cells

To elaborate the underlying molecular mechanisms of the different cellular responses to IL-6 trans- and classic signalling we made use of the specific trans-signalling inducer Hy-IL-6. Hy-IL-6 is a fusion protein of IL-6 and sIL-6Rα [14]. We verified the applicability of Hy-IL-6 to induce trans-signalling in lieu of IL-6 and sIL-6Rα. Using the law of mass action and considering the dissociation constant K_D_ = 0.5 nM of the IL-6:IL-6Rα complex [48], we calculated how many IL-6:sIL-6Rα complexes are formed for a given number of IL-6 and sIL-6Rα. Next, we compared signalling induced by either IL-6:sIL-6Rα complex or an equimolar amount of Hy-IL-6. HepG2 cells, that express both gp130 and membrane-bound IL-6Rα, were stimulated for 30 min with 0.17 nM IL-6 + 100 nM sIL-6Rα, forming 0.17 nM IL-6:sIL-6Rα complex. For comparison HepG2 cells were stimulated with 0.17 nM Hy-IL-6 or left untreated. Phosphorylation of STAT3 was analysed by intracellular flow cytometry. No difference in STAT3 activation in response to trans-signalling induced by either Hy-IL-6 or the IL-6:sIL-6Rα complex was obvious (Additional file 1: Figure S1). Hence, Hy-IL-6 can act as a substitute for equimolar amounts of IL-6:sIL-6Rα complexes.

We next investigated whether the strength of IL-6-induced STAT3 activation in response to classic signalling is different to that in response to trans-signalling. We therefore compared the kinetics of STAT3 phosphorylation, SOCS3 mRNA and SOCS3 protein expression in HepG2 cells stimulated with IL-6 (0.08 nM (Fig. 1a) or 0.17 nM (Fig. 1b)) to initiate classic signalling, or with Hy-IL-6 (0.08 nM (Fig. 1a) or 0.17 nM (Fig. 1b)) to initiate trans-signalling. STAT3 phosphorylation and SOCS3 protein expression were analysed by quantitative Western blotting (Additional file 1: Figure S6) and SOCS3 mRNA was quantified by qRT-PCR. IL-6-induced trans- and classic signalling result in transient phosphorylation of STAT3. However, trans-signalling-induced STAT3 activation (red) is more pronounced than classic signalling-induced STAT3 phosphorylation (blue). SOCS3 mRNA and protein expression follow the peak of STAT3 phosphorylation. Both SOCS3 mRNA and protein expression are higher in response to trans-signalling than to classic signalling (Fig. 1).

In summary, STAT3 phosphorylation is markedly elevated in response to trans-signalling compared to classic signalling, resulting also in higher SOCS3 mRNA and protein expression levels.

### Set-based modelling supports the hypothesis that intracellular signalling initiated by IL-6 trans- or classic signalling does not differ

So far, it is not understood whether the observed differences of STAT3 activation in response to trans- and classic signalling (Fig. 1) are caused by different strength of receptor activation or by different mechanisms and dynamics of signalling downstream to receptor activation. To test these alternative hypotheses we made use of the described set-based modelling and model validation approach [43]. This method enables testing whether a model can reasonably be parameterised despite uncertain experimental data. In other words, set-based modelling allows invalidating a model that is not capable of reproducing given experimental data, and therefore to invalidate the underlying model hypothesis (Fig. 2 AI, blue line). Notably, the solution of a set-based model relies on a feasibility problem (FP) and several mathematical relaxation steps (Fig. 2 AII, Additional file 1: Text S1). These relaxation steps lead to the identification of valid parameter sets. These sets, however, may contain false positive parameters that result in model trajectories which do not match to experimental data (Fig. 2 AIII). Strictly speaking, only a model invalidation, but not a model validation is possible [43]. Once a model is found to be not invalid, an exclusion of false positive parameter sets is performed using an outer-bounding algorithm (Fig. 2 AIII). Furthermore, analyses such as Monte Carlo sampling are applied to determine valid parameterisations resulting in trajectories that match the experimental data (orange cross and trajectory Fig. 2 AIII).

Our goal was to test the hypothesis that topologies and kinetics downstream of receptor activation are identical in trans- and classic signalling. To do so, we used set-based modelling. We considered three different dynamic computational models (Fig. 2b). While the first model combines trans- and classic signalling, the second and third models describe trans- and classic signalling by two separated models (see Material and Methods for modelling assumptions, Additional file 1: Text S2.1 and Additional file 1: Tables S1 and S2 for detailed model descriptions). We modelled the specifics of classic signalling by considering that IL-6 first binds to IL-6Rα followed by binding of the IL-6:IL-6Rα complex to gp130. In case of trans-signalling Hy-IL-6 associates directly with gp130.

To validate/invalidate the according hypotheses, we developed the following workflow (Fig. 2c). First, parameter estimation is performed for the three initial models (Fig. 2b) using set-based parameter estimation. Subsequently, the estimated parameter ranges serve as inputs to reduced models lacking SOCS3 negative feedback. The aim of this second step is to further confine the initial parameter ranges using again set-based parameter estimation. Next, the results of both set-based parameter estimation rounds are merged which results in calibrated models with reduced parameter ranges.

To finally test whether our calibrated models cannot be invalidated and hence support our initial hypothesis, a yes/no workflow is applied (Fig. 2c). Notably, the results of the yes/no workflow applied in this study are depicted in bold black arrows in Fig. 2c, while alternative workflows are given by dotted arrows. We first ask whether the obtained parameter ranges for the three calibrated models (Fig. 2c box 1) overlap. In case the ranges are disjoint, the initial hypothesis is deemed invalid. In case the parameter ranges overlap, we next ask whether the model combining both, trans- and classic signalling, yields the smallest and the same parameter ranges as at least one of the models describing trans-signalling only or classic signalling only (box 2). If this question is neglected, a Monte Carlo sampling analysis is subsequently performed for all three models to check whether at least individual parameter sets can be found that overlap between all three models (boxes 2a and b). In case the parameter sets are disjoint, we can state that our initial hypothesis is invalid. If in contrast, the obtained parameter sets overlap, we deem this hypothesis as not invalid.

Above, we asked whether the model which combines both, trans- and classic signalling yields the same parameter ranges as the models describing only trans-signalling and only classic signalling. If this applies, we can state that the model which combines both, trans- and classic signalling constrains parameter ranges best (Fig. 2c box 3) and can be used for further Monte Carlo sampling analyses (box 3a). If finally, parameterisations are determined, such that the model is capable to represent all experimental data (box 3b), we cannot invalidate the hypothesis that trans- and classic signalling-induced Jak/STAT signalling employ the same pathway topology downstream of receptor activation (box 3c). Subsequently, the developed and not invalid model can be used for further analyses, while in negative case, the hypothesis is deemed invalid and the model is rejected.

To perform the proposed workflow, we employed the experimental data presented in Fig. 1. Additionally, we determined the number of IL-6Rα and gp130 molecules on the cell surface by a bead-based FACS assay as 2099 ± 347 molecules/cell surface (2.2 nM ± 0.4 nM) and 16,198 ± 2965 molecules/cell surface (16.8 nM ± 3.1 nM), respectively (Fig. 2d, Additional file 1: Figure S5). Furthermore, the absolute number of STAT3 in HepG2 cells was measured by quantitative Western blotting as 9.2 × 10^5^ ± 4.2 × 10^5^ molecules/cell (958 nM ± 445 nM), (Fig. 2d and Additional file 1: Figure S2). Based on the algebraic eqs. (S6)–(S8) presented in Additional file 1: Text S2.1.1 the amounts determined for STAT3, IL-6Rα and gp130 were used as start values to calculate the model quantities of the [IL-6:IL-6Rα] complex, of the non-activated hexameric receptor complex [Rcomplex], and of unphosphorylated STAT3 [STAT3]. Furthermore, initial boundaries of all parameters were specified in a global range of 10^− 9^-10^3^ covering all biologically-justified parameter values (Additional file 1: Table S3). We further considered the range of 0.5–50 nM for the dissociation constant of the IL-6:IL-6Rα complex (K_D1_ = $\frac{p_2}{p_1}$) [11, 49–52] and the range of 0.01–0.05 nM for the dissociation constants of the IL-6:IL-6Rα:gp130 complex $\left({\mathrm{K}}_{\mathrm{D}2}=\frac{{\mathrm{p}}_4^{\mathrm{cl}}}{{\mathrm{p}}_3^{\mathrm{cl}}}\right)$ and of the Hy-IL-6:gp130 complex $\left({\mathrm{K}}_{\mathrm{D}3}=\frac{{\mathrm{p}}_4^{\mathrm{tr}}}{{\mathrm{p}}_3^{\mathrm{tr}}}\right)$ [11, 50] (Fig. 2b).

Based on these constraints, we used our set-based parameter estimation workflow (Fig. 2c) to test whether the hypothesis that intracellular signalling mechanisms do not differ between trans- and classic signalling. We started with the initial model that combines both, trans- and classic signalling. The first round of set-based modelling provided restrictions on the model parameters p_3_^tr^, p_4_^tr^_,_ and p_7_-p_12,_ while other parameters could not (p_5_, p_6_ and p_13_) or only marginally be restricted (p_1_, p_2,_ p_3_^cl^, p_4_^cl^; Fig. 2e dark grey bars compared to initial parameter intervals in green and Additional file 1: Table S3, fifth column). Of note, none of the parameter sets was found to be empty so that the model could not be invalidated at this step.

To further validate the results, we additionally implemented the model into the Data2Dynamics software package [44], and performed identifiability analyses. As result, parameters for which we estimated tight outer bounds (p_3_^tr^, p_4_^tr^_,_ p_9_-p_12_) were identifiable in contrast to the remaining parameters. Hence, this different approach supports the results from set-based modelling.

Including both, trans- and classic signalling simultaneously in a single model may constrain the parameter boundaries in comparison to specific models on trans- and classic signalling. Thus, in a second step, we separated trans- and classic signalling in two models and used set-based modelling to estimate parameter ranges for the two models individually. The results for parameter estimation of these two models are depicted in Fig. 2e (blue bars for classic signalling only; red bars for trans-signalling only) and Additional file 1: Table S4. Again, all parameter sets were found to be non-empty. We could restrict 14 (p_1_, p_2,_ p_3_^cl^, p_4_^cl^, p_3_^tr^, p_4_^tr^, p_7_, p_8_, p_9_, p_delay1_, p_10_, p_11_, p_delay2_, p_12_) out of 17 model parameter ranges. However, for the remaining three parameters (p_5_, p_6_ and p_13_) no restrictions could be made (Fig. 2e).

In summary the performed set-based parameter estimation did not render our models invalid and enabled us to restrict most of the unknown parameters. This result counts for all three initial models.

### Decoupling of fast and slow processes within Jak/STAT signalling improves parameter restriction

**Fig. 3:**
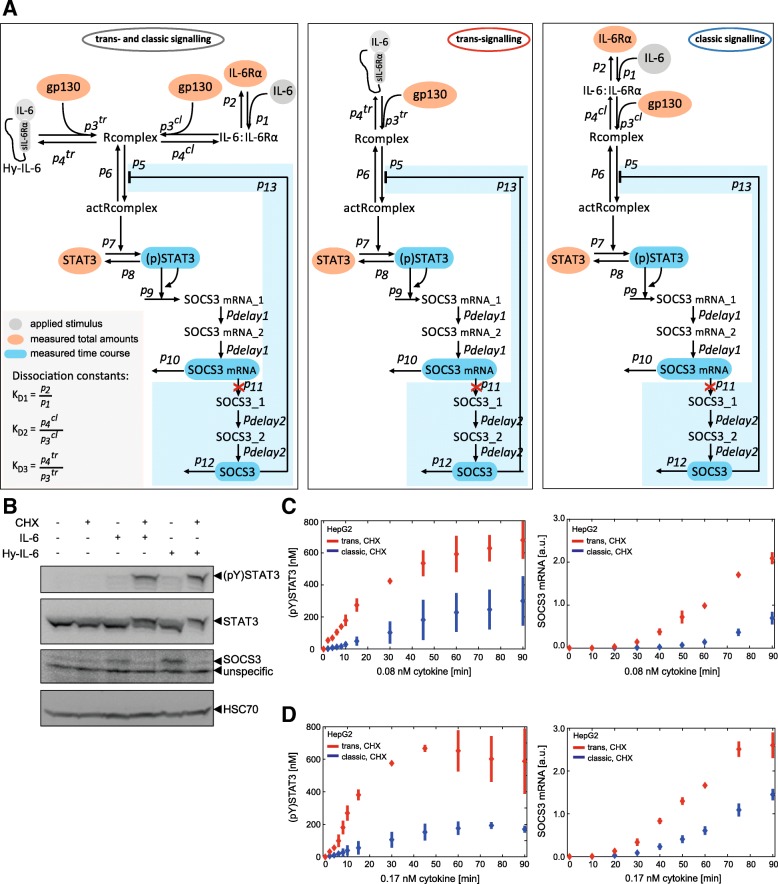
Network topologies of the reduced models and additional experimental data for improving model calibration. **a** Network topology of the reduced ODE models disregarding SOCS3 protein expression (red crosses) and negative feedback by SOCS3. The light blue boxes depict the network parts which are disregarded in the reduced models by setting the corresponding parameter values to zero. **b** HepG2 cells were pretreated with or without cycloheximide for 30 min and subsequently stimulated with IL-6 (0.42 nM) and Hy-IL-6 (0.17 nM), respectively. STAT3 phosphorylation and STAT3, SOCS3, and HSC70 protein expression were evaluated by Western blotting. STAT3 and HSC70 expression served as loading control. Representative results of *n* = 3 independent experiments are shown. HepG2 cells were pretreated with cycloheximide for 30 min and subsequently stimulated with 0.08 nM (**c**) or 0.17 nM (**d**) IL-6 (blue) and Hy-IL-6 (red). STAT3 phosphorylation and expression of STAT3 protein were evaluated by Western blotting. Expression of STAT3 served as loading control. The expression of SOCS3 mRNA was analysed using qRT-PCR. Diamonds correspond to the mean and bars to the STD of *n* = 3 experiments. Data normalization was performed as described in Additional file 1: Text S3

The so far unrestricted parameters are important to describe the initial and fast activation of the pathway. Thus, we analysed these model parameters in reduced models that decouple the early and fast receptor activation from the subsequent slow reactions, which include the synthesis of SOCS3 protein and the SOCS3-dependent negative feedback. We exploit the fact that biochemical parameters are independent of the network topology and used the estimated parameter ranges obtained by analysing the initial models (Fig. 2e, Additional file 1: Table S3, fifth column and Additional file 1: Table S4, second and fifth columns) as input values for the reduced models (Fig. 2c). As before, one reduced model described trans- and classic signalling and two additional reduced models described either trans- or classic signalling (Fig. 3a). By setting the parameters p_11_, p_delay2_, p_12,_ and p_13_ to zero we assumed the production of SOCS3 protein - and hence the resulting negative feedback - to be blocked (blue part in Fig. 3a). To match these assumptions experimentally, we analysed the kinetics of Jak/STAT signalling in HepG2 cells while blocking the synthesis of SOCS3 protein with cycloheximide (CHX). CHX blocked IL-6 and Hy-IL-6-induced SOCS3 protein expression and consequently cytokine-induced STAT3 phosphorylation was strongly increased (Fig. 3b). For kinetic analyses HepG2 cells were stimulated with IL-6 or Hy-IL-6 (0.08 nM and 0.17 nM) in the presence of CHX (Fig. 3c, d). Whereas IL-6 and Hy-IL-6-induced phosphorylation of STAT3 and SOCS3 mRNA expression were transient in the absence of CHX (Fig. 1), phosphorylation of STAT3 was sustained in the presence of CHX and reached a plateau after 60 min of stimulation (Fig. 3c, d left panels and Additional file 1: Figure S7). Cytokine-induced expression of SOCS3 mRNA rose continuously until the end of the experiment (Fig. 3c, d right panels). Notably, also in the absence of the SOCS3 feedback, trans-signalling was stronger than classic signalling.

**Fig. 4:**
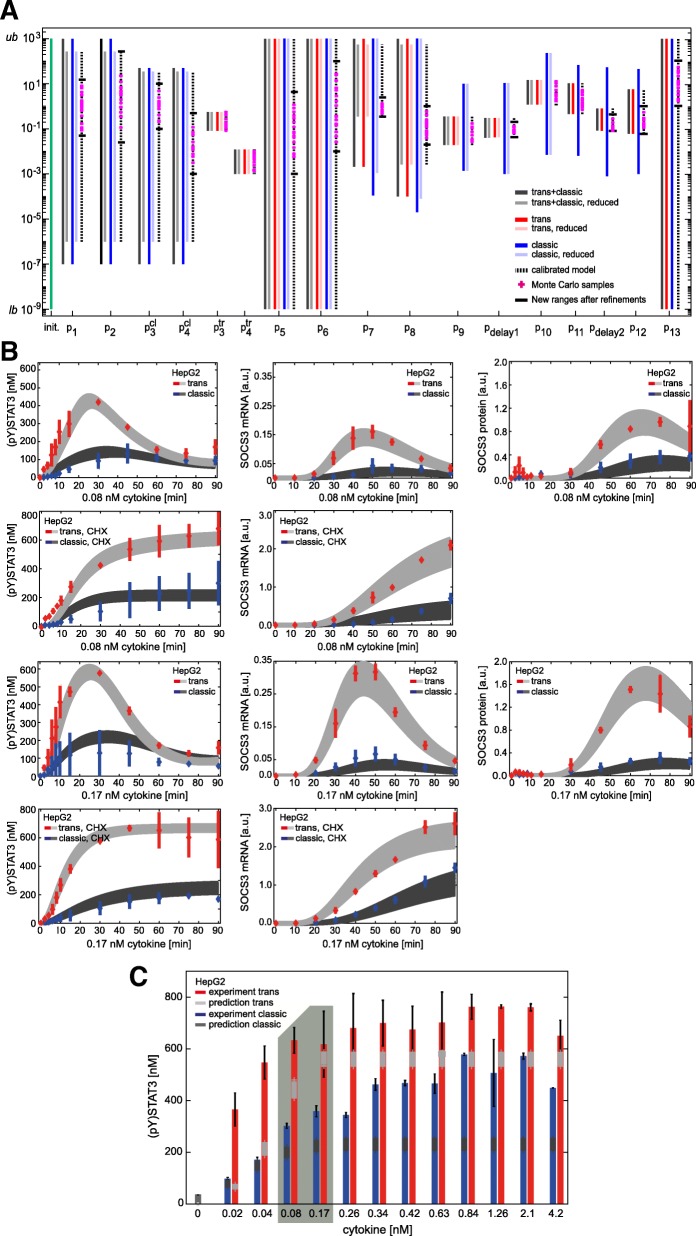
Improved parameterisation and refinement of set-based parameter estimation, based on Monte Carlo sampling. **a** Results for outer-bounding of model parameters. Initial parameter bounds (green bar) range from 10^− 9^ (lower bound, *lb*) to 10^3^ (upper bound, *ub*). Dark grey, blue and red bars depict parameter ranges for the individual parameters after set-based analyses of the initial models. Light grey, light blue and light red bars depict parameter ranges after parameter estimation of the reduced models disregarding the SOCS3-mediated feedback. Dotted bars show final set-based estimation results for the calibrated model. Magenta plus signs depict exemplary valid Monte Carlo samples and black horizontal lines show the newly obtained parameter ranges after model refinements. **b** 150 out of 150,000 Monte Carlo samples that yield the lowest quadratic distance between model predictions and experimental data and reasonable represent all experimental data available. Model outputs (light and dark grey corridors) were plotted against experimental data (red and blue) presented in Figs. 1 and 4. **c** Model predictions for dose-dependent phosphorylation of STAT3 in response to 30 min classic (dark grey) and trans-signalling (light grey) based on the 150 Monte Carlo samples from (A). HepG2 cells were stimulated with indicated amounts of IL-6 (blue bars) or Hy-IL-6 (red bars). STAT3 phosphorylation was evaluated by intracellular flow cytometry using specific fluorescent antibodies against STAT3 (p)Y705. For independent experiments mean fluorescence of 10^4^ cells per cytokine concentration was calculated. Data are given as mean ± STD from *n* = 3 experiments. The grey box depicts experimental conditions used in Fig. 1 (stimulation with 0.08 nM and 0.17 nM cytokine for 30 min)

These additional experimental data were used for parameter estimations of the reduced models. Compared to the analyses of the initial models, ranges of parameters p_1_, p_2_, p_3_^cl^, p_4_^cl^, p_7,_ and p_8_ could be further reduced for all three models lacking the SOCS3 feedback loop (Fig. 4a, compare light colours (w/o SOCS3 feedback) with the corresponding dark colours (with SOCS3 feedback); Additional file 1: Table S3 sixth column and Additional file 1: Table S4 third and sixth columns). Notably, ranges for parameters p_5_, p_6,_ and p_13_ could neither be restricted using the initial models and corresponding data, nor using the reduced models with the additional data.

We next merged the results from first and second set-based parameter estimation results by choosing the smallest obtained parameter ranges from both rounds and thereby obtained so called calibrated models (Fig. 2c, Additional file 1: Table S3, seventh column and Additional file 1: Table S4, fourth and seventh columns).

Subsequently, we followed the flow chart as given in Fig. 2c to test (non-)invalidity of our initial hypothesis, that signalling mechanisms downstream of receptor activation do not differ between trans- and classic signalling. The obtained parameter sets for the calibrated models describing trans- and classic signalling, trans-signalling only, and classic signalling overlap (compare grey, blue and red bars in Fig. 4a) (Fig. 2c box 1). Furthermore, the parameter ranges estimated from the model describing only trans-signalling yielded the same ranges than from the model describing both, trans- and classic signalling (compare grey and red bars in Fig. 4a) (Fig. 2c box 2). In contrast, parameter ranges estimated from the model describing only classic signalling were less restricted than parameter ranges estimated from the two other models. Hence, neither the individual model for trans- nor the individual model for classic signalling enable further parameter restrictions than the combined model. Consequently, we concluded that the model which combines both, trans- and classic signalling, constrains the parameter ranges best and can be used for further analyses (Fig. 2c box 3).

### Monte Carlo sampling and set-based refinements of parameter ranges

Using the set-based modelling approach, the ranges of the parameters of the calibrated model that describes both, trans- and classic signalling were restricted optimally using set-based modelling and the given experimental data (Fig. 4a, dotted bars). However, to finally test whether we can (in)validate our initial hypothesis, we analysed whether defined parameter sets within the restricted parameter ranges exist that enable us to reproduce our experimental data (Fig. 2c box 3). We therefore applied Monte Carlo sampling to the calibrated model that describes both, trans- and classic signalling (Fig. 2c box 3a). The estimated parameter ranges (Fig. 4a, dotted bars) served as outer bounds for Monte-Carlo sampling. Out of 150,000 parameterisations, we derived the 150 parameterisations with lowest square deviation between our model predictions and the experimental data (Fig. 4a, Additional file 1: Table S3, seventh column). Exemplary parameterisations out of these 150 parameterisations are depicted as magenta crosses in Fig. 4a. These 150 parameterisations allowed predictions, which are in line with the experimental data (Fig. 2c box 3a; Fig. 4b). Specifically, in Fig. 4b model predictions for the kinetics of trans- and classic signalling-induced STAT3 phosphorylation, SOCS3 mRNA expression, and SOCS3 protein expression for up to 90 min are depicted in dark and light grey corridors, respectively. These corridors result from simulations of the model with the determined 150 best parameterisations. Experimental data (as shown in Figs. 1 and 3c,d) are given in red for trans-signalling and blue for classic signalling. As the model was capable to represent all experimental data using the obtained parameterisations, we could not invalidate the model. Thus, we were not forced to reject our initial hypothesis that signalling mechanisms downstream of receptor activation do not differ between trans- and classic signalling (Fig. 2c box 3c).

As can be seen in Fig. 4a for most of the parameters, the parameterisations derived by Monte Carlo sampling did not cover the complete parameter ranges restricted by set-based parameter estimation. This, however, is no proof for the non-existence of valid parameter solutions in other regions of the restricted parameter ranges. That is why, we next confirmed our results from Monte Carlo sampling independently. Specifically, we aimed to demonstrate, that regions where no samples were determined by Monte Carlo sampling are indeed invalid, i.e. do not contain parameters that sufficiently describe the experimental data. To do so, we used an iterative procedure that is built upon successive refinements of the lower and upper parameter bounds (Additional file 1: Table S3, seventh column). Starting with parameter p_1_, we moved the previously estimated lower and upper bounds (Fig. 4a, dotted bars) of p_1_ inwards, while testing at each step if the model is deemed invalid. By this, we obtained refined and tightened parameter bounds for p_1_. Of note, boundaries for p_2_ were automatically restricted after refining p_1_ as the ratio of p_1_ to p_2_ represents the dissociation constant of the IL-6:IL-6Rα complex. We proceeded with parameter p_3_^cl^ similar as to p_1_. As result, also parameter p_4_^cl^ could be further restricted. The procedure was repeated for the remaining parameters resulting in a further refinement of the estimated parameter ranges depicted as black horizontal lines in Fig. 4a (Additional file 1: Table S3, seventh column in brackets). These refined ranges comprised all determined parameterisations. Of note, all results from Monte Carlo sampling are within the refined parameter ranges. Thus, our results from Monte Carlo sampling could be confirmed.

In summary, Monte Carlo sampling and a subsequent refinement of parameter ranges allowed us to develop a mathematical model of IL-6-induced trans- and classic signalling with tight and valid parameter ranges that sufficiently reproduced experimental data.

We finally challenged the predictive capacity of our model. We therefore calculated the dose-dependent phosphorylation of STAT3 expected after 30 min of stimulation with either IL-6 or Hy-IL-6. For experimental validation, we stimulated HepG2 cells with 13 different equimolar concentrations of Hy-IL-6 and IL-6 for 30 min and monitored STAT3 phosphorylation by intracellular flow cytometry. As control we included the experimental conditions used in Fig. 4b (stimulation with 0.08 nM and 0.17 nM cytokine for 30 min). Both, trans- and classic signalling induced a dose-dependent phosphorylation of STAT3. Notably, trans-signalling was stronger than classic signalling for all cytokine concentrations tested. Model predictions were in line with these experimental results, that were not used for parameterisation, which proves the predictive power of our calibrated model (Fig. 4c).

In summary, we established a parameterised predictive computational model that describes the differences between trans- and classic signalling without proposing differences in canonical intracellular signalling.

### Model prediction reveals that differences between trans- and classic signalling are caused by the ratio of gp130 to IL-6Rα on the cell surface

**Fig. 5:**
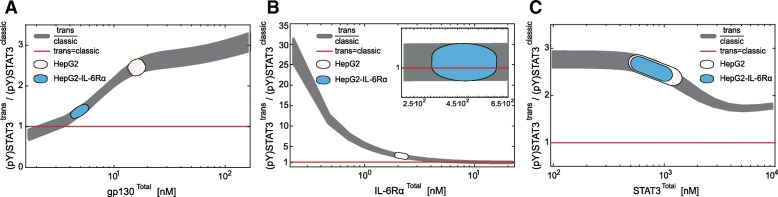
Ratio of IL-6Rα to gp130 expression on the cell surface determines strength of trans- and classic signalling. Model-based prediction of the ratio of trans- to classic signalling-induced STAT3 phosphorylation after 30 min stimulation with IL-6 and Hy-IL-6 (0.17 nM). **a** Expression of gp130 was predefined to range between 1.68 nM and 168 nM while expression of IL-6Rα^Total^ (2.2 nM) and STAT3^Total^ (958 nM) were fixed. **b** Expression of IL-6Rα was predefined to range between 0.22 nM and 22.2 nM while expression of gp130^Total^ (16.8 nM) and STAT3^Total^ (958 nM) were fixed. **c** Expression of STAT3 was predefined to range between 95.8 nM and 9580 nM while expression of gp130^Total^ (16.8 nM) and IL-6Rα^Total^ (2.2 nM) were fixed. Grey corridors correspond to model predictions. Red line depicts equal strength of trans- and classic signalling. The white areas represent the range of gp130 (A), IL-6Rα (B) and STAT3 (C) expression in HepG2 cells, respectively. The blue areas represent the range of gp130 (A), IL-6Rα (B) and STAT3 (C) expression in HepG2-IL-6Rα cells, respectively

As we could not invalidate the hypothesis that topology and kinetics of Jak/STAT signalling downstream of receptor activation are the same for trans- and classic signalling, we next asked which components of the signalling pathway are responsible for the observed differences in STAT3 activation in response to trans- and classic signalling. To analyse whether the amount of receptors on the cell surface affects the ratio of classic to trans-signalling we varied the start values of gp130 and membrane-bound IL-6Rα. With these different input variables we performed model predictions using the obtained 150 best Monte Carlo parameter samples and our final predictive calibrated model for trans- and classic signalling. We predicted the ratio of trans-signalling to classic signalling-induced STAT3 phosphorylation after 30 min of cytokine stimulation as model output. A ratio of 1 implies that both signalling modes lead to equally strong STAT3 activation (red line in Fig. 5) whereas a ratio > 1 indicates that trans-signalling is stronger than classic signalling.

The mean concentrations of IL-6Rα^Total^, STAT3^Total^ and gp130 were determined as 2.2 nM, 958 nM and 16.9 nM, respectively (see Fig. 2d). First, we fixed IL-6Rα^Total^ and STAT3^Total^ to their mean concentrations and varied the mean value of gp130^Total^ ± one order of magnitude (1.69 nM to 169 nM, Fig. 5a). Notably, for endogenous gp130 concentrations (white area) the model well rendered the high ratio of trans- to classic signalling shown experimentally. For increasing amounts of gp130 the ratio of trans- to classic signalling further increased, while the ratio of trans- to classic signalling decreased for lower amounts of gp130.

Next, we kept the concentration of gp130^Total^ and STAT3^Total^ constant but varied the amount of IL-6Rα^Total^ ± one order of magnitude (0.22 nM to 22 nM, Fig. 5b). The white area depicts the concentration of endogenous IL-6Rα ± STD. As shown experimentally trans-signalling was two to three times stronger than classic signalling under these conditions. Interestingly, for higher concentrations of IL-6Rα^Total^ the difference between STAT3 phosphorylation during trans- and classic signalling was completely ablated.

From these observations we concluded that the ratio of IL-6Rα to gp130 on the cell surface crucially determines the strength of STAT3 activation in response to trans- and classic signalling. Limited expression of membrane-bound IL-6Rα restricts STAT3 activation in response to classic signalling but does not limit trans-signalling because soluble IL-6Rα compensates for limited expression of membrane-bound IL-6Rα. If gp130 expression is lower than IL-6Rα expression, gp130 acts as a bottleneck for trans- and classic signalling, hence, trans-signalling cannot surpass classic signalling. In line with this hypothesis HepG2 cells, where trans-signalling is stronger than classic signalling, express eight times more gp130 than IL-6Rα (Fig. 2d).

The results from modelling do not argue for classic or trans-signalling-specific signal transduction downstream of the respective activated receptor complex. To substantiate this hypothesis, we applied our model to predict the influence of the extent of STAT3 expression on the ratio of trans- to classic signalling-induced STAT3 activation. We predicted STAT3 phosphorylation for STAT3^Total^ ranging from 95.8 nM to 9580 nM (Fig. 5c) and fixed expression of IL-6Rα^Total^ and gp130^Total^ to endogenous amounts. As shown before, STAT3^Total^ concentrations in the range of endogenous STAT3 expression are predicted to result in STAT3 phosphorylation, that is two to three times stronger in response to trans-signalling than in response to classic signalling. With increasing amounts of STAT3^Total^ this difference decreased slightly. Yet, STAT3 phosphorylation in response to trans-signalling was still two times stronger than in response to classic signalling – even at a concentration of 9580 nM STAT3^Total^. This supports our hypothesis that intracellular signalling is not causative for the differences between trans- and classic signalling.

In summary, our model analyses let us hypothesize that the ratio of gp130 to IL-6Rα determines the differences between trans- and classic signalling.

### Experimental validation of the impact of receptor ratios on the differences between trans- and classic signalling

**Fig. 6:**
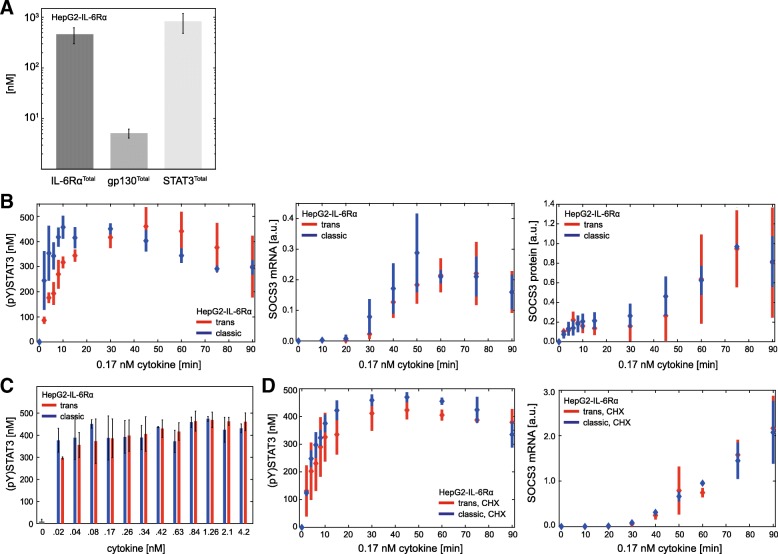
High IL-6Rα/gp130 receptor ratio in HepG2-IL-6Rα cells ablates difference between trans- and classic signalling. **a** Expression of gp130 and IL-6Rα on the surface of HepG2-IL-6Rα cells was quantified by flow cytometry using QIFIKIT. Mean ± STD values from *n* = 4 independent experiments are shown. Expression of STAT3 in HepG2-IL-6Rα cells was quantified by Western blotting using recombinant calibrator proteins. Mean ± STD values from n = 7 independent experiments are shown. **b** HepG2-IL-6Rα cells were stimulated with IL-6 (blue) and Hy-IL-6 (red) (0.17 nM). STAT3 phosphorylation and expression of STAT3, SOCS3, and HSC70 protein were evaluated by Western blotting. Expression of STAT3 and HSC70 served as loading control. The expression of SOCS3 mRNA was analysed using qRT-PCR. Diamonds correspond to the mean and bars to the STD of n = 4 experiments. Data normalization was performed as described in Additional file 1: Text S3. **c** HepG2-IL-6Rα cells were stimulated with indicated amounts of IL-6 (blue bars) or Hy-IL-6 (red bars). STAT3 phosphorylation was evaluated by intracellular flow cytometry using specific fluorescent antibodies against STAT3 (p)Y705. For independent experiments mean fluorescence of 10^4^ cells per cytokine concentration was calculated. Data are given as mean ± STD from n = 3 experiments. **d** HepG2-IL-6Rα cells were pretreated with cycloheximide for 30 min and subsequently stimulated with 0.17 nM IL-6 (blue) and Hy-IL-6 (red), respectively. STAT3 phosphorylation and STAT3 expression were evaluated by Western blotting. STAT3 expression served as loading control. The expression of SOCS3 mRNA was analysed using qRT-PCR. Diamonds correspond to the mean and bars to the standard deviation for n = 3 independent experiments. Data normalization was performed as described in Additional file 1: Text S3

To challenge our hypothesis that the receptor ratio determines the difference between trans- and classic signalling, we set up additional experiments in HepG2 cells that stably overexpress IL-6Rα (HepG2-IL-6Rα) on the cell surface. We quantified IL-6Rα and gp130 expression by FACS analysis (Fig. 6a). In contrast to HepG2 cells, that express approximately eight times more gp130 than IL-6Rα (Fig. 2d), HepG2-IL-6Rα cells express approximately 100-times more IL-6Rα than gp130. Of note, STAT3 expression in HepG2 and HepG2-IL-6Rα cells was similar (Fig. 2d and Fig. 6a; Fig. 5c blue area). According to our model predictions HepG2-IL-6Rα cells reflect a situation in which the ratio of classic to trans-signalling equals to one (Fig. 5a and b blue areas). To validate these predictions experimentally, we stimulated HepG2-IL-6Rα cells with IL-6 or Hy-IL-6 (0.17 nM) and analysed STAT3 phosphorylation, SOCS3 mRNA expression, and protein expression in response to trans- and classic signalling (Fig. 6b, Additional file 1: Figure S8A). In accordance with model predictions there is no difference between trans- and classic signalling in HepG2-IL-6Rα cells. Furthermore, HepG2-IL-6Rα cells were stimulated with 13 different equimolar concentrations of Hy-IL-6 and IL-6 for 30 min. Dose-dependent STAT3 phosphorylation was equal in response to trans- and classic signalling (Fig. 6c). Finally, HepG2-IL-6Rα cells were stimulated with IL-6 or Hy-IL-6 (0.17 nM) and treated with CHX for blocking SOCS3 protein synthesis. STAT3 phosphorylation as well as SOCS3 mRNA expression were similar in response to trans- and classic signalling also in the absence of SOCS3 feedback (Fig. 6d, Additional file 1: Figure S8B).

In summary, in line with the prediction by our model, trans- and classic signalling in HepG2-IL-6Rα cells resulted in equal kinetics and dose-dependent activation of Jak/STAT signalling. Data-driven modelling and experimental data revealed that the stronger activity of IL-6-induced trans-signalling in HepG2 cells can sufficiently be explained by the ratio of gp130 to IL-6Rα on the cell surface.

### Strength of trans- and classic signalling translates into strength of cell proliferation

**Fig. 7:**
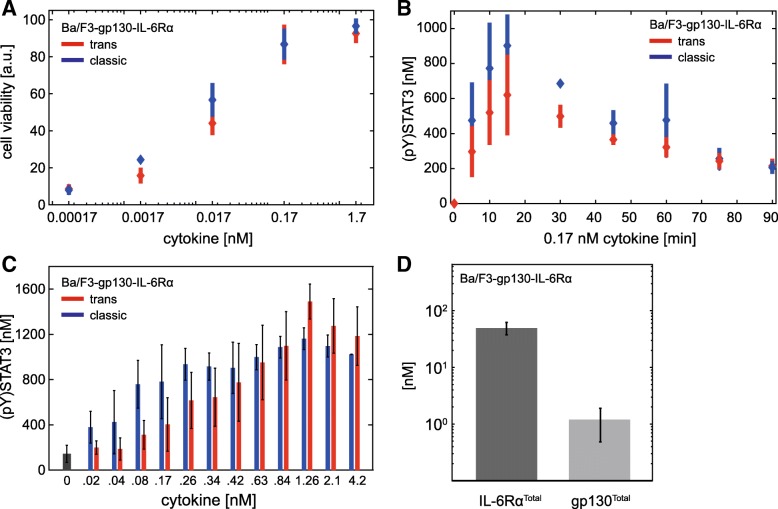
Trans- and classic signalling-induced growth of Ba/F3-gp130-IL-6Rα cells do not differ. **a** Ba/F3-gp130-IL-6Rα cells were stimulated with IL-6 (blue) or Hy-IL-6 (red) as indicated. After 48 h of incubation cell growth was measured using Cell Titer Blue reagent. Diamonds correspond to the mean and bars to the standard deviation for *n* = 4 experiments. **b** Ba/F3-gp130-IL-6Rα cells were stimulated with IL-6 (blue) and Hy-IL-6 (red) (0.17 nM). STAT3 phosphorylation and STAT3 expression were evaluated by Western blotting. STAT3 expression served as loading control. Diamonds correspond to the mean and bars to the standard deviation for *n* = 4 independent experiments. Data normalization was performed as described in Additional file 1: Text S3. **c** Ba/F3-gp130-IL-6Rα cells were stimulated with the indicated amounts of IL-6 (blue bars) or Hy-IL-6 (red bars), respectively. STAT3 phosphorylation was evaluated by intracellular flow cytometry using specific fluorescent antibodies against STAT3 (p)Y705. For independent experiments mean fluorescence of 10^4^ cells per cytokine concentration was calculated. Data are given as mean ± STD from *n* = 3 experiments. **d** The expression of gp130 and IL-6Rα at the surface of Ba/F3-gp130-IL-6Rα cells was analysed by flow cytometry using QIFIKIT. Mean ± STD from *n* = 4 independent experiments is shown

So far, our analyses focussed on IL-6-induced signalling, but have not investigated the downstream effects of STAT3 activation which, besides others, result in proliferation of blood cells [53]. Murine pre B cells (Ba/F3) are a convenient and established cellular system for studying cytokine-induced proliferation. Ba/F3 cells proliferate in response to IL-3. Ba/F3 cells stably expressing gp130 and IL-6Rα proliferate in response to IL-6 [45]. Thus, to determine how trans- and classic signalling translate into cell proliferation, we analysed trans- and classic signalling-induced proliferation of Ba/F3-gp130-IL-6Rα cells. The cells were treated with increasing equimolar amounts of Hy-IL-6 and IL-6 to induce trans- and classic signalling, respectively. Proliferation was analysed after 48 h and found to be equal in response to trans- and classic signalling (Fig. 7a). To test whether this correlates with equal dynamics of cellular signalling in response to trans- and classic signalling, we analysed the kinetics of Jak/STAT signalling in response to 0.17 nM Hy-IL-6 or IL-6 (Fig. 7b, Additional file 1: Figure S9). In response to both stimuli, STAT3 phosphorylation increases up to 15 min and subsequently decreases slowly to a steady state reached about 75 min post start of stimulation. Dose-dependent STAT3 phosphorylation in response to stimulation with either IL-6 or Hy-IL-6 for 30 min is documented in Fig. 7c. Again, no differences in IL-6- and Hy-IL-6-induced STAT3 phosphorylation were obvious.

These results correspond to equal IL-6 trans- and classic signalling-induced STAT3 activation observed in HepG2-IL-6Rα cells. Based on these similarities and our modelling results (Fig. 5) we hypothesized that Ba/F3-gp130-IL-6Rα cells express more IL-6Rα than gp130. Indeed, quantifying the number of IL-6Rα and gp130 molecules in Ba/F3-gp130-IL-6Rα revealed 40 times more IL-6Rα than gp130 molecules on the cell surface (Fig. 7d). These results further strengthen the hypothesis that differences between trans- and classic signalling are primarily caused by the IL-6Rα to gp130 ratio.

### Pharmacological inhibition of intracellular IL-6-induced signalling does not discriminate between trans- and classic signalling

**Fig. 8:**
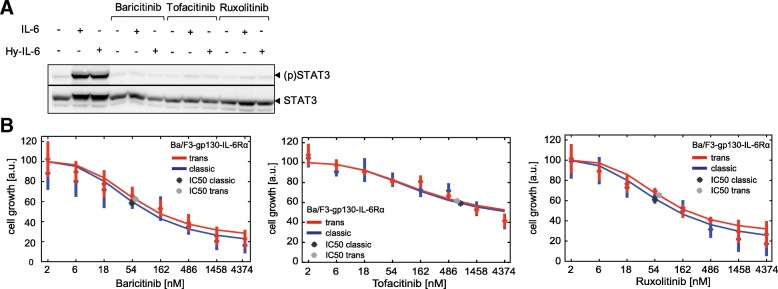
Pharmacological inhibition of Jak does not discriminate between trans- and classic signalling. **a** Ba/F3-gp130-IL-6Rα cells were pretreated with or without the indicated inhibitors (5 μM) for 30 min and subsequently stimulated with 10 ng/ml IL-6 (0.42 nM) or Hy-IL-6 (0.17 nM). STAT3 phosphorylation and STAT3 expression were evaluated by Western blotting. STAT3 expression served as loading control. Representative results of *n* = 3 independent experiments are shown. **b** Ba/F3-gp130-IL-6Rα cells were incubated with IL-6 (0.42 pM) or Hy-IL-6 (0.52 pM) with the indicated concentrations of the corresponding inhibitors for 48 h. Cell growth was measured using Cell Titer Blue reagent. Diamonds correspond to the mean and bars to the STD of *n* = 3 experiments. Red and blue lines indicate a maximum likelihood 4 parameter logistic regression. The determined IC50 values are given as dark stars for classic signalling and light grey stars for trans-signalling

IL-6-induced trans-signalling is associated with severe inflammatory diseases, whereas classic signalling contributes to the anti-inflammatory activities of IL-6 [3, 4]. These observations have encouraged the development of approaches to specifically block trans-signalling. We aimed to test, whether three Jak inhibitors (Baricitinib, Ruxolitinib, Tofacitinib) [54] differentially block IL-6-induced trans- and classic signalling. We demonstrated that the inhibitors interfere with STAT3 activation by applying the inhibitors to cells stimulated with IL-6 or Hy-IL-6 (Fig. 8a). To compare their inhibitory potential against IL-6-induced trans- and classic signalling, IC50 values for inhibition of trans- and classic signalling-induced growth of Ba/F3-gp130-IL-6Rα cells were determined (Fig. 8b). All three inhibitors inhibited growth of Ba/F3-gp130-IL-6Rα cells in a dose-dependent manner. However, none of the Jak inhibitors discriminated between cell proliferation initiated by IL-6 classic or trans-signalling.

From these experiments, we conclude that interfering in the first step of intracellular signalling is not appropriate to specifically target trans-signalling. These findings further strengthen the hypothesis that differences observed between IL-6-induced trans- and classic signalling are not caused by differences in intracellular signalling. Instead, the response towards IL-6 trans- and IL-6 classic signalling is crucially determined by the ratio of IL-6 receptor α to gp130 expression.

## Discussion

We employed a data-driven computational modelling-supported systems biology approach, based on a combination of set-based parameter estimation with experimental analyses to demonstrate that differences between IL-6-induced trans- and classic signalling are caused by the ratio of the expression of IL-6-receptor subunits on the cell surface. If the amount of gp130 exceeds the amount of IL-6Rα, trans-signalling is stronger than classic signalling. In contrast, if IL-6Rα expression exceeds expression of gp130, both pathways activate downstream signalling equally strong.

Most somatic cells do not express IL-6Rα on their cell surface and, hence, are not responsive to classic signalling. Yet, cells that express membrane-bound IL-6Rα respond to both trans- and classic signalling. For these cell types the availabilities of IL-6, membrane-bound IL-6Rα, soluble IL-6Rα, and gp130 determine the response towards IL-6 trans- and classic signalling. Changes in the expression of these molecules allow fine-tuning of the cellular response. Different expression of IL-6 and soluble IL-6Rα in healthy and diseased individuals has been extensively demonstrated. In inflammatory conditions serum concentration of soluble IL-6Rα increases up to five times [28, 55]. Of note, at the site of inflammation the availability of soluble IL-6Rα is further enhanced. Increased availability of soluble IL-6Rα in combination with a strong inflammation-induced increase (> 100 fold) in IL-6 broadens the spectrum of IL-6-responsive cells but also increases the strength of the cellular response towards IL-6. In particular, hepatocytes are prominent IL-6-target cells which express significantly more gp130 than IL-6Rα. Thus, the increase of soluble IL-6Rα would have decisive effects for these cells.

In the body, IL-6-induced responses further achieve regulatory complexity by the presence of soluble gp130 that – in contrast to soluble IL-6Rα – is an antagonist of IL-6-induced signalling [12]. Specifically, soluble gp130 together with soluble IL-6R is usually discussed to constitute a buffer system that, in the excess of soluble IL-6Rα, favours trans-signalling and in the excess of sgp130, blocks IL-6-induced signalling. Initially, sgp130 was described as a specific inhibitor of trans-signalling [12]. However, at high concentrations, sgp130 also blocks classic signalling by forcing free IL-6 into IL-6:IL-6Rα:sgp130 complexes [56]. Our study emphasises that the differential potential for inhibition of trans- and classic signalling by soluble gp130 and also the buffering capacity of sgp130 are controlled by the ratio of gp130 to IL-6Rα expression.

Several approaches have been developed to interfere with disease-associated IL-6-induced signalling [54]. The humanized anti-IL-6Rα antibody Tocilizumab is approved to treat rheumatoid arthritis and juvenile idiopathic arthritis in more than 100 countries [57]. Beside the success of Tocilizumab some patients do not respond to treatment with Tocilizumab for yet unknown reasons, whereas other patients develop an increased susceptibility against bacterial infections [54]. This latter by-effect is commonly explained by the fact that blockade of IL-6Rα inhibits both trans- and classic signalling. To circumvent this, strategies to specifically block trans-signalling without affecting classic signalling are currently under way. To specifically block pro-inflammatory trans-signalling, a first trans-signalling specific inhibitor (sgp130Fc, Olamkicept) that utilizes the antagonistic action of naturally occurring sgp130 on IL-6-induced trans-signalling was developed [12, 58]. Sgp130Fc was successfully applied in animal models of Crohn’s disease [58] and is currently in phase II clinical trials for treatment of IBD in general and ulcerative colitis in particular [54]. However, here we confirm indications from previous studies of others [13, 56, 59] that the stoichiometry of the proteins involved in receptor activation and inhibition crucially determines signalling strength. Therefore, the success of sgp130-based specific inhibition of trans-signalling will probably greatly improve from personalized treatment strategies. Indeed, expression of soluble IL-6Rα, IL-6, and endogenous sgp130 differ strongly from patient to patient and are affected by disease state and genotype [60]. Thus, the exact quantification of the expression of these components will be necessary for personalized treatments. Currently, methods to determine the concentration of free and complexed soluble IL-6Rα, IL-6, and sgp130 in human serum are being developed [49]. Specifically, the application of computational models to predict signalling strength and regulation from individual expression levels will help to guide personalized application of specific trans-signalling inhibitors.

Beside strategies to interfere with IL-6 receptor activation inhibitors to alter intracellular signalling are also currently approved or in clinical trials [54]. Here, we have tested whether Jak inhibitors that are already approved to treat rheumatoid arthritis and Crohn’s disease (Tofacitinib), myelofibrosis and polycythaemia vera (Ruxolitinib) or rheumatoid arthritis (Baricitinib) differentially block trans- and classic signalling. In line with our observation that canonical intracellular Jak/STAT signalling does not differ between trans- and classic signalling, these three inhibitors do not differentially affect the two signalling modes and thus are not applicable for a specific therapeutic inhibition of IL-6 trans-signalling. Furthermore, these inhibitors are not pathway specific, yet affect other Jak-dependent cytokine signalling pathways.

Whereas our study was focussed on activation of Jak/STAT signalling recent work has highlighted that the activation of IL-6-induced PI3K/AKT and MEK/ERK pathways are also differentially regulated by trans- and classic signalling [61]. The study of Zegeye et al. reports that in endothelial cells PI3K/AKT and MEK/ERK pathways are solely activated via IL-6 trans-signalling whereas Jak/STAT signalling is activated by both, classic and trans-signalling [61]. No statement on how the MEK/ERK pathway is affected by trans- and classic signalling in HepG2 cells can be made, since HepG2 cells exhibit constitutive ERK1/2 phosphorylation (data not shown). However, Hy-IL-6 induces a transient activation of ERK1/2 in HepG2-IL-6Rα cells. IL-6 classic signalling does not activate this pathway in HepG2-IL-6Rα cells (data not shown). Further analyses will clarify the involvement of the receptor subunit ratios in differential activation of these pathways.

## Conclusion

In summary, our data and model-based computational modelling analyses highlight the dependency of IL-6-induced Jak/STAT signalling on the quantitative availabilities and ratios of the receptor subunits involved in activation of Jak/STAT signalling. These results emphasize the necessity to develop individualized and computer-aided approaches to interfere with disease-associated signalling.

## Additional file

Additional file 1: **Text S1.** Set-based model invalidation and parameter estimation. **Text S2.** Mathematical modelling. **Text S3.** Normalization of experimental data. **Table S1.**: Expression and description of considered model fluxes. **Table S2.** Description of state variables and initial conditions. **Table S3.** Description of model parameters, units, initial uncertainty intervals and set-based estimation results for the model describing classic and trans-signalling. **Table S4.** Set-based estimation results for models describing either classic or trans-signalling, respectively. **Figure S1.** Hy-IL-6 simulates IL-6-induced trans-signalling. **Figure S2.** Quantification of STAT3 and (p)STAT3. **Figure S3.** Validation of the specificity of the fluorescent antibody against STAT3 (p)Y705. **Figure S4.** Dose-dependent Hy-IL-6-induced STAT3 phosphorylation using Western Blotting. **Figure S5.** Quantification of gp130 and IL-6Rα cell surface expression. **Figure S6.** Raw data of Figure 1a and b. **Figure S7.** Raw data of Figure 3c and d. **Figure S8.** Raw data of Figure 6b and d **Figure S9.** Raw data of Figure 7b. (PDF 1204 kb)

## Abbreviations

CHX: Cycloheximide
FP: Feasibility problem
gp130: Glycoprotein 130
Hy-IL-6: Hyper IL-6
IBD: Inflammatory bowel disease
IL-6: Interleukin-6
IL-6Rα: Interleukin-6 receptor α
Jak: Janus kinase
LP: Linear program
ODE: Ordinary differential equation
RA: Rheumatoid arthritis
SDHA: Succinate dehydrogenase complex A
sgp130: Soluble gp130
sIL-6Rα: Soluble IL-6Rα
SOCS3: Suppressor of cytokine signalling 3
STAT3: Signal transducer and activator of transcription 3
STD: standard deviation

## Authors’ information

FS chairs the Department of Systems Biology of the Institute of Biology at the Otto-von-Guericke University, Magdeburg, Germany. AD is an academic staff member, HR and UB are PhD students and HC was a bachelor student in the Department of Systems Biology.

RF chairs the Laboratory for Systems Theory and Control of the Institute for Automation Engineering at the Otto-von-Guericke University Magdeburg, Germany. EB is an academic staff member and NR is a PhD student in the Laboratory for Systems Theory and Control.

HJH and MSB contributed during their work as a Senior Scientists at the Laboratory for Systems Theory and Control of the Institute for Automation Engineering at the Otto-von-Guericke University Magdeburg, Germany. HJH is now a principal scientist and team leader at Boehringer-Ingelheim.

SS is head of the Automatic Control and System Dynamics Laboratory at the Chemnitz University of Technology, Chemnitz, Germany.
