# Supplementary material for: Response to IL-6 trans- and IL-6 classic signalling is determined by the ratio of the IL-6 receptor α to gp130 expression: fusing experimental insights and dynamic modelling
Source: Cell Commun Signal. 2019 May 17;17:46. doi: 10.1186/s12964-019-0356-0 (PMC6525395; doi:10.1186/s12964-019-0356-0)
Supplement: Supplementary file 1 — Text S1. Set-based model invalidation and parameter estimation. Text S2. Mathematical modelling. Text S3. Normalization of experimental data. Table S1.: Expression and description of considered model fluxes. Table S2. Description of state variables and initial conditions. Table S3. Description of model parameters, units, initial uncertainty intervals and set-based estimation results for the model describing classic and trans-signalling. Table S4. Set-based estimation results for models describing either classic or trans-signalling, respectively. Figure S1. Hy-IL-6 simulates IL-6-induced trans-signalling. Figure S2. Quantification of STAT3 and (p)STAT3. Figure S3. Validation of the specificity of the fluorescent antibody against STAT3 (p)Y705. Figure S4. Dose-dependent Hy-IL-6-induced STAT3 phosphorylation using Western Blotting. Figure S5. Quantification of gp130 and IL-6Rα cell surface expression. Figure S6. Raw data of Figure 1a and b. Figure S7. Raw data of Figure 3c and d. Figure S8. Raw data of Figure 6b and d Figure S9. Raw data of Figure 7b. (PDF 1204 kb) [file 12964_2019_356_MOESM1_ESM.pdf]

Response to IL-6 trans- and IL-6 classic signalling is determined by the ratio of the IL-6 receptor α to gp130 expression: fusing experimental insights and dynamic modelling

Heike Reeh[1*], Nadine Rudolph[2*], Ulrike Billing[1], Henrike Christen[1], Stefan Streif[2,3], Eric Bullinger[2], Monica Schliemann-Bullinger[2], Rolf Findeisen[2], Fred Schaper[1], Heinrich J. Huber [2,4] and Anna Dittrich[1+]

[1] Department of Systems Biology, Institute of Biology, Faculty of Natural Sciences, Otto-von-Guericke University Magdeburg, Universitätsplatz 2, 39106 Magdeburg, Germany

[2] Department of Systems Theory and Automatic Control, Institute for Automation Engineering, Faculty of Electronics Engineering and Information Technology, Otto-von-Guericke University Magdeburg, Universitätsplatz 2, 39106 Magdeburg, Germany

[3] Automatic Control and System Dynamics Laboratory, Institute of Automation, *Chemnitz University* of *Technology*, Reichenhainer Straße 70, 09107 Chemnitz, Germany

[4] Target Discovery Research, Boehringer Ingelheim Pharmaceutical Research and Development Centre Biberach, Birkendorfer Straße 65, 88397 Biberach, Germany

[*]These authors contributed equally, [+] Corresponding author

Supplementary Material

Table of content

S1. Set-based model invalidation and parameter estimation ... 3

S1.1 Formulation of a feasibility problem ... 3

S1.2 Semidefinite and linear relaxations ... 4

S1.3 Outer-bounding of parameters ... 4

S2. Mathematical modelling ... 5

S2.1 Model description ... 5

S2.1.1 Conserved moieties ... 8

S3. Normalization of experimental data ... 14

S3.1 Western blot ... 14

S3.2 qRT-PCR ... 15

S4. Supplementary Figures ... 17

S5. References ... 27

S1. Set-based model invalidation and parameter estimation

To apply the set-based modelling approach, a discrete-time approximation of the considered continuous-time model (Table S1) was derived. We here applied a first order Euler discretisation scheme resulting in the model

$$\left.\begin{aligned} &f(x(k+1), x(k), u(k), p) = 0 \\ &h(y(k), x(k), u(k), p) = 0. \end{aligned}\right\} \quad \text{(S1)}$$

In (S1), f and h are polynomial or rational difference equations and $x(k), u(k), y(k)$ and p denote the model states, inputs, outputs and parameters. These variables are equivalent to the continuous-time system, but bounded by semialgebraic sets instead of continuous equality and inequality constraints.

S1.1 Formulation of a feasibility problem

To formulate the model invalidation and estimation task, the following feasibility problem (FP) was set up:

$$\text{FP:} \begin{cases} \quad \text{find} & \xi \\ \text{subject to} & \mathrm{g_i}(\xi) \end{cases} \quad \text{(S2)}$$

where $\xi \in \mathbb{R}^{n_\xi}$ is a vector that contains all time-variant and time-invariant variables from (S1). The constraints $\mathrm{g_i}(\xi)$ represent the nonlinear dynamics in (S1) as well as the set-based uncertainties (lower and upper bounds of all model variables including initial conditions from Table S2 and bounds of the parameters as given in Table S3).

The general idea of the set-based approach is to check whether the FP admits a solution or not, which reflects the capability of model (S1) to satisfy the given constraints. Due to nonlinearities in our model equations (Table S1) and possible non-convex parameter sets, the

FP became non-convex and was thus, hard to solve. As remedy, the FP was relaxed as explained next.

S1.2 Semidefinite and linear relaxations

The relaxation (or convexification) of the above problem (S2) required several steps, which we describe shortly in the following. For technical details of the relaxation steps, we refer to [1-3]. The basic idea of relaxation of (S2) was to replace the nonlinearities with simpler expressions, which could then be solved much more efficiently. In a first relaxation step, the FP was relaxed into a convex semidefinite program (SDP). However, since our models for IL-6-induced classic and trans-signalling are very large (up to 1335 variables), the SDP was further relaxed into a linear program (LP). Notably, since the original non-convex problem (S2) is always contained in the relaxed problem, we do not miss any solution. Thus, each solution of (S2) is also one of the SDP and LP, however, not vice-versa.

S1.3 Outer-bounding of parameters

The above relaxation approach was employed to tackle the set-based estimation problem allowing to check infeasibility of our model and to approximate the unknown parameter sets [1-3]. The algorithm to derive such approximations performed an outer-bounding of the parameter sets by sequentially and iteratively tightening the lower and upper bounds of the single parameters. In more detail, the feasibility problem (S2) was replaced by an optimization problem in which the single parameter bounds were minimized or maximized, respectively. As result, a boxed-shaped outer approximation of the parameters was determined.

S2. Mathematical modelling

S2.1 Model description

The differential equations describing IL-6-induced classic and trans-signalling are given by the following equations:

$d[\text{IL-6:IL-6R}\alpha]/dt = v_1 - v_2 - 2v_{3a} + 2v_{4a}$

$d[\text{gp130}]/dt = 2v_{4a} - 2v_{3a}$ (classic), $d[\text{gp130}]/dt = 2v_{4b} - 2v_{3b}$ (trans)

$d[\text{actRcomplex}]/dt = v_5 - v_6$

$d[\text{(p)STAT3}]/dt = v_7 - v_8$

$d[\text{SOCS3 mRNA\_1}]/dt = v_9 - v_{10}$

$d[\text{SOCS3 mRNA\_2}]/dt = v_{10} - v_{11}$

$d[\text{SOCS3 mRNA}]/dt = v_{11} - v_{12}$

$d[\text{SOCS3\_1}]/dt = v_{13} - v_{14}$

$d[\text{SOCS3\_2}]/dt = v_{14} - v_{15}$

$d[\text{SOCS3}]/dt = v_{15} - v_{16}$

The descriptions of the flux expressions v_i can be extracted from Table S1 and description of all model states including initial conditions for set-based analyses can be taken from Table S2.

Table S1: Expression and description of considered model fluxes

Flux	Equations	Description
v_1	$p_1 \cdot [IL\text{-}6] \cdot [IL\text{-}6R\alpha]$	Association of IL-6 and IL-6Rα
v_2	$p_2 \cdot [IL\text{-}6{:}IL\text{-}6R\alpha]$	Dissociation of IL-6:IL-6Rα
v_3		Association of hexameric receptor complex
v_{3a}	$p_3^{cl} \cdot [IL\text{-}6{:}IL\text{-}6R\alpha]^2 \cdot [gp130]^2$	classic $(IL\text{-}6{:}IL\text{-}6R\alpha{:}gp130)_2$
v_{3b}	$p_3^{tr} \cdot [Hy\text{-}IL\text{-}6]^2 \cdot [gp130]^2$	trans $(Hy\text{-}IL\text{-}6{:}gp130)_2$
v_4		Dissociation of hexameric receptor complex
v_{4a}	$p_4^{cl} \cdot [Rcomplex]$	classic
v_{4b}	$p_4^{tr} \cdot [Rcomplex]$	trans
v_5	$\frac{p_5 \cdot [Rcomplex]}{1 + p_{13} \cdot [SOCS3]}$	Receptor complex activation and negative feedback inhibition to inhibit receptor activity
v_6	$p_6 \cdot [actRcomplex]$	Receptor complex deactivation
v_7	$p_7 \cdot [actRcomplex] \cdot [STAT3]$	STAT3 phosphorylation
v_8	$p_8 \cdot [(p)STAT3]$	STAT3 dephosphorylation
v_9	$p_9 \cdot [(p)STAT3]^2 \cdot (1nM + [(p)STAT3])$	Initiation of SOCS3 mRNA transcription
v_{10}	$p_{delay1} \cdot [SOCS3\ mRNA\_1]$	Transcriptional delay
v_{11}	$p_{delay1} \cdot [SOCS3\ mRNA\_2]$	Transcriptional delay
v_{12}	$p_{10} \cdot [SOCS3\ mRNA]$	SOCS3 mRNA degradation
v_{13}	$p_{11} \cdot [SOCS3\ mRNA]$	Initiation of SOCS3 protein synthesis
v_{14}	$p_{delay2} \cdot [SOCS3\_1]$	SOCS3 protein synthesis delay
v_{15}	$p_{delay2} \cdot [SOCS3\_2]$	SOCS3 protein synthesis delay

v_{16}	$p_{12} \cdot [SOCS3]$	SOCS3 protein degradation

Table S2: Description of state variables and initial conditions

**State variable**	**Description**	**Initial condition [unit]**
IL-6, Hy-IL-6	Free Interleukin-6, Hyper-IL-6	According to experiment, [nM]
IL-6Rα	Membrane-bound IL-6Rα	[1.8,2.6] [nM]
gp130	Membrane-bound gp130	[13.8,19.9] [nM]
IL-6:IL-6Rα	Dimerized receptor complex classic signalling	0 [nM]
Rcomplex	Non-active hexameric receptor complex	0 [nM]
actRcomplex	Active hexameric receptor complex	0 [nM]
STAT3	Signal transducer and activator of transcription 3	[513,1403] [nM]
(p)STAT3	Phosphorylated STAT3	0 [nM]
SOCS3 mRNA<br>SOCS3 mRNA_1<br>SOCS3 mRNA_2	Suppressor of cytokine signalling 3 mRNA and intermediate states _1 and _2	0 [a.u.]<br>0 [a.u.]<br>0 [a.u.]
SOCS3<br>SOCS3_1<br>SOCS3_2	Suppressor of cytokine signalling 3 and intermediate states _1 and _2	0 [a.u.]<br>0 [a.u.]<br>0 [a.u.]

S2.1.1 Conserved moieties

Concentrations for the quantities [IL-6:IL-6Rα], [Rcomplex] and [STAT3] (see Table S1) can be derived from the following algebraic equations:

$$[\text{IL-6}\alpha^{\text{Total}}] = [\text{IL-6R}\alpha] + [\text{IL-6:IL-6R}\alpha] + 2[\text{Rcomplex}] + 2[\text{actRcomplex}] \quad \text{(S6)}$$

$$[\text{gp130}^{\text{Total}}] = [\text{gp130}] + 2[\text{Rcomplex}] + 2[\text{actRcomplex}] \quad \text{(S7)}$$

$$[\text{STAT3}^{\text{Total}}] = [\text{STAT3}] + [\text{(p)STAT3}]. \quad \text{(S8)}$$

In (S6)-(S8), the total amounts of the proteins are set according to the experimentally determined mean values ± standard deviation (see Figure 2D in the main text).

S2.2 Parameter estimation

For set-based analyses and outer-bounding of the parameters, the ODE-system was discretised and subsequently implemented in ADMIT [3]. Descriptions of all parameters and set-based estimation results are given in Table S3.

Table S3: Description of model parameters, units, initial uncertainty intervals and set-based estimation results for the model describing classic and trans-signalling. $\underline{p}_i$ and $\overline{p}_i$ indicate the estimated lower and upper bounds for the individual parameters using an outer-bounding algorithm (Text S1.3). The first and the second columns depict the corresponding parameter name and its unit, the third column gives a description of the parameter and the fourth column depicts the initial chosen uncertainty intervals. The fifth, sixth and seventh columns depict the set-based parameter estimation results for the initial model (Figure 2B), the reduced model (Figure 3A) and the calibrated model, respectively. Parameter ranges obtained after iterative refinements based on Monte Carlo sampling are depicted in brackets (seventh column).

Par.	Unit	Description	Initial uncertainty intervals	$[\underline{\mathbf{p}}_\mathbf{i}, \overline{\mathbf{p}}_\mathbf{i}]$ initial model	$[\underline{\mathbf{p}}_\mathbf{i}, \overline{\mathbf{p}}_\mathbf{i}]$ reduced model	$[\underline{\mathbf{p}}_\mathbf{i}, \overline{\mathbf{p}}_\mathbf{i}]$ calibrated model
p_1	nM^{-1} min^{-1}	Association of IL-6:IL-6Rα complex	$[10^{-9},10^{3}]$	$[10^{-7},9.9\cdot10^{2}]$	$[10^{-6},2.7\cdot10^{2}]$	$[10^{-6},2.7\cdot10^{2}]$ ($[5\cdot10^{-2},1.5\cdot10^{1}]$)
p_2	min^{-1}	Dissociation of IL-6:IL-6Rα complex	$[10^{-9},10^{3}]$ $\frac{p_2}{p_1}$ = 0.5-50 [nM] [4, 5]	$[10^{-7},9.9\cdot10^{2}]$	$[10^{-6},2.7\cdot10^{2}]$	$[10^{-6},2.7\cdot10^{2}]$ ($[2.5\cdot10^{-3},2.7\cdot10^{2}]$)
p_3^{cl}	nM^{-3} min^{-1}	Association of $(IL\text{-}6{:}IL\text{-}6R\alpha{:}gp130)_2$ complexes	$[10^{-9},10^{3}]$	$[10^{-7},5\cdot10^{1}]$	$[10^{-6},3.5\cdot10^{1}]$	$[10^{-6},3.5\cdot10^{1}]$ ($[10^{-1}, 10^{1}]$)
p_3^{tr}	nM^{-3} min^{-1}	Association of $(Hy\text{-}IL\text{-}6{:}gp130)_2$ complexes	$[10^{-9},10^{3}]$	$[8.3\cdot10^{-2},5.5\cdot10^{-1}]$	$[8.3\cdot10^{-2},5.5\cdot10^{-1}]$	$[8.3\cdot10^{-2},5.5\cdot10^{-1}]$

p_4^{cl}	min^{-1}	Dissociation of (IL-6:IL-6Rα:gp130)$_2$ complexes	$[10^{-9},10^{3}]$	$[10^{-7},5\cdot10^{1}]$	$[10^{-6},3.5\cdot10^{1}]$	$[10^{-6},3.5\cdot10^{1}]$ ($[10^{-3},5\cdot10^{-1}]$)
p_4^{tr}	min^{-1}	Dissociation of (Hy-IL-6:gp130)$_2$ complexes	$[10^{-9},10^{3}]$ $\frac{p_4}{p_3} =$ 0.01-0.05 [nM] [4, 6]	$[9.9\cdot10^{-4},10^{-2}]$	$[9.9\cdot10^{-4},10^{-2}]$	$[9.9\cdot10^{-4},10^{-2}]$
p_5	min^{-1}	Activation of the receptor complex	$[10^{-9},10^{3}]$	$[10^{-9},10^{3}]$	$[10^{-9},10^{3}]$	$[10^{-9},10^{3}]$ ($[10^{-3},5]$)
p_6	min^{-1}	Deactivation of the receptor complex	$[10^{-9},10^{3}]$	$[10^{-9},10^{3}]$	$[10^{-9},10^{3}]$	$[10^{-9},10^{3}]$ ($[10^{-2},10^{2}]$)
p_7	nM^{-1} min^{-1}	Phosphorylation of STAT3	$[10^{-9},10^{3}]$	$[2\cdot10^{-3},9.9\cdot10^{2}]$	$[3.5\cdot10^{-1},5.5\cdot10^{2}]$	$[3.5\cdot10^{-1},5.5\cdot10^{2}]$ ($[3.5\cdot10^{-1},5]$)
p_8	min^{-1}	Dephosphorylation of STAT3	$[10^{-9},10^{3}]$	$[4\cdot10^{-4},9.9\cdot10^{2}]$	$[2.6\cdot10^{-3},5.5\cdot10^{2}]$	$[2.6\cdot10^{-3},5.5\cdot10^{2}]$ ($[2\cdot10^{-2},1]$)
p_9	μM^{-3} min^{-1}	Transcription of SOCS3 mRNA	$[10^{-9},10^{3}]$	$[1.9\cdot10^{-2},3.5\cdot10^{-1}]$	no improvement	$[1.9\cdot10^{-2},3.5\cdot10^{-1}]$
p_{delay1}	min^{-1}	Transcriptional delay	$[10^{-9},10^{3}]$	$[4\cdot10^{-2},3\cdot10^{-1}]$	no improvement	$[4\cdot10^{-2},3\cdot10^{-1}]$

						($[4\cdot10^{-2},2\cdot10^{-1}]$)
p_{10}	min^{-1}	Degradation of SOCS3 mRNA	$[10^{-9},10^{3}]$	$[0.12\cdot10^{1},1.4\cdot10^{1}]$	no improvement	$[0.12\cdot10^{1},1.4\cdot10^{1}]$
p_{11}	min^{-1}	Translation of SOCS3 protein	$[10^{-9},10^{3}]$	$[4.5\cdot10^{-1},10^{1}]$	-	$[4.5\cdot10^{-1},10^{1}]$
p_{delay2}	min^{-1}	Translational delay	$[10^{-9},10^{3}]$	$[8\cdot10^{-2},8\cdot10^{-1}]$	-	$[8\cdot10^{-2},8\cdot10^{-1}]$<br>($[8\cdot10^{-2},3\cdot10^{-1}]$)
p_{12}	min^{-1}	Degradation of SOCS3 protein	$[10^{-9},10^{3}]$	$[6\cdot10^{-2},5.64]$	-	$[6\cdot10^{-2},5.64]$<br>($[6\cdot10^{-2},1]$)
p_{13}	$a.u.^{-1}$	Negative feedback inhibition	$[10^{-9},10^{3}]$	$[10^{-9},10^{3}]$	-	$[10^{-9},10^{3}]$<br>($[1, 300]$)

Table S4: Set-based estimation results for models describing either classic or trans-signalling, respectively. $\underline{p}_i$ and $\overline{p}_i$ indicate the estimated lower and upper bounds for the individual parameters using an outer-bounding algorithm (Text S1.3). The first column describes the corresponding parameter name. The second, third and fourth columns depict the set-based parameter estimation results for the initial (Figure 2B, right), the reduced (Figure 3A, right) and the calibrated model describing classic signalling. The fifth, sixth and seventh columns depict the set-based parameter estimation results for the initial (Figure 2B, middle), the reduced (Figure 3A, middle) and the calibrated model describing trans-signalling.

**Par.**	$[\underline{p}_i, \overline{p}_i]$ **initial model, classic signalling**	$[\underline{p}_i, \overline{p}_i]$ **reduced model, classic signalling**	$[\underline{p}_i, \overline{p}_i]$ **calibrated model, classic signalling**	$[\underline{p}_i, \overline{p}_i]$ **initial model, trans-signalling**	$[\underline{p}_i, \overline{p}_i]$ **reduced model, trans- signalling**	$[\underline{p}_i, \overline{p}_i]$ **calibrated model, trans-signalling**
p_1	$[10^{-7}, 9.9^2]$	$[10^{-6}, 2.7 \cdot 10^2]$	$[10^{-6}, 2.7 \cdot 10^2]$	-	-	-
p_2	$[10^{-7}, 9.9^2]$	$[10^{-6}, 2.7 \cdot 10^2]$	$[10^{-6}, 2.7 \cdot 10^2]$	-	-	-
p_3^{cl}	$[10^{-7}, 5 \cdot 10^1]$	$[10^{-6}, 3.5 \cdot 10^1]$	$[10^{-6}, 3.5 \cdot 10^1]$	-	-	-
p_3^{tr}	-	-	-	$[8.3 \cdot 10^{-2}, 5.5 \cdot 10^{-1}]$	$[8.3 \cdot 10^{-2}, 5.5 \cdot 10^{-1}]$	$[8.3 \cdot 10^{-2}, 5.5 \cdot 10^{-1}]$
p_4^{cl}	$[10^{-7}, 5 \cdot 10^1]$	$[10^{-6}, 3.5 \cdot 10^1]$	$[10^{-6}, 3.5 \cdot 10^1]$	-	-	-
p_4^{tr}	-	-	-	$[9.9 \cdot 10^{-4}, 10^{-2}]$	$[9.9 \cdot 10^{-4}, 10^{-2}]$	$[9.9 \cdot 10^{-4}, 10^{-2}]$
p_5	$[10^{-9}, 10^3]$	$[10^{-9}, 10^3]$	$[10^{-9}, 10^3]$	$[10^{-9}, 10^3]$	$[10^{-9}, 10^3]$	$[10^{-9}, 10^3]$
p_6	$[10^{-9}, 10^3]$	$[10^{-9}, 10^3]$	$[10^{-9}, 10^3]$	$[10^{-9}, 10^3]$	$[10^{-9}, 10^3]$	$[10^{-9}, 10^3]$

p_7	$[1.1\cdot10^{-4},10^3]$	$[2\cdot10^{-3},9.9\cdot10^2]$	$[2\cdot10^{-3},9.9\cdot10^2]$	$[2\cdot10^{-3},9.9\cdot10^2]$	$[3.5\cdot10^{-1},5.5\cdot10^2]$	$[3.5\cdot10^{-1},5.5\cdot10^2]$
p_8	$[2\cdot10^{-5},10^3]$	$[8\cdot10^{-5},9.9\cdot10^2]$	$[8\cdot10^{-5},9.9\cdot10^2]$	$[4\cdot10^{-4},9.9\cdot10^2]$	$[2.6\cdot10^{-3},5.5\cdot10^2]$	$[2.6\cdot10^{-3},5.5\cdot10^2]$
p_9	$[1.3\cdot10^{-2},10^1]$	no improvement	$[1.3\cdot10^{-2},10^1]$	$[1.9\cdot10^{-2},3.5\cdot10^{-1}]$	no improvement	$[1.9\cdot10^{-2},3.5\cdot10^{-1}]$
p_{delay1}	$[10^{-3}, 1.08\cdot10^1]$	no improvement	$[10^{-3},1.08\cdot10^1]$	$[4\cdot10^{-2},3\cdot10^{-1}]$	no improvement	$[4\cdot10^{-2},3\cdot10^{-1}]$
p_{10}	$[7\cdot10^{-3}, 20.3\cdot10^1]$	no improvement	$[7\cdot10^{-3},20.3\cdot10^1]$	$[0.12\cdot10^1,1.4\cdot10^1]$	no improvement	$[0.12\cdot10^1,1.4\cdot10^1]$
p_{11}	$[6.4\cdot10^{-3},6.7\cdot10^1]$	-	$[6.4\cdot10^{-3},6.7\cdot10^1]$	$[4.5\cdot10^{-1},10^1]$	-	$[4.5\cdot10^{-1},10^1]$
p_{delay2}	$[8\cdot10^{-4},5.3\cdot10^1]$	-	$[8\cdot10^{-4},5.3\cdot10^1]$	$[8\cdot10^{-2},8\cdot10^{-1}]$	-	$[8\cdot10^{-2},8\cdot10^{-1}]$
p_{12}	$[10^{-3},4.4\cdot10^1]$	-	$[10^{-3},4.4\cdot10^1]$	$[6\cdot10^{-2},5.64]$	-	$[6\cdot10^{-2},5.64]$
p_{13}	$[10^{-9},10^3]$	-	$[10^{-9},10^3]$	$[10^{-9},10^3]$	-	$[10^{-9},10^3]$

S3. Normalization of experimental data

S3.1 Western blot

All experiments were performed in a minimum of three independent biological replicates as indicated in the figure captions. Numerical values for protein expression and activation were obtained by quantification of signals obtained from Western blot analyses. Values of the respective proteins were divided by those of the loading controls of the same sample. Data for each individual experiment were normalized to the value reached at t = 30 min (analysis of STAT3 phosphorylation) or t = 60 min (analysis of SOCS3 expression) post stimulation. The resulting value at t = 0 min was regarded as background and thus subtracted from the values for all time points within an individual experiment. Mean values and standard deviation over all biological replicates were calculated.

STAT3 phosphorylation induced in response to different cytokine stimuli was monitored by separated Western blots. For the sake of comparability cells were stimulated with 0.08 nM and 0.17 nM Hy-IL-6 (HepG2) or IL-6 (HepG2-IL-6Rα, Ba/F3-gp130-IL-6Rα) for 30 min and STAT3 phosphorylation was analysed by Western blotting at the same Western Blot (n = 3 each). The ratio of STAT3 phosphorylation induced by both concentrations of the cytokine, i.e. $r^{pSTAT3} = \frac{\mathrm{pSTAT3\ (0.17nM)}}{\mathrm{pSTAT3\ (0.08nM)}}$ was calculated for the mean of three independent replicates. The resulting ratio was used to normalize STAT3 phosphorylation detected on separated blots for cells stimulated with 0.08 nM and 0.17 nM cytokine. This allows comparison of the results from different experiments.

Obtained values for relative STAT3 activation were converted to absolute numbers using values from quantitative immunoprecipitation (Figure S2).

SOCS3 expression induced in response to different cytokine stimuli, was monitored by separated Western blots. For the sake of comparability HepG2 and HepG2-IL-6Rα cells were stimulated with 0.08 nM and 0.17 nM Hy-IL-6 for 60 min and SOCS3 expression was analysed by Western blotting at the same Western Blot (n = 3 each). The ratio of SOCS3 expression induced by both concentrations of Hy-IL-6, i.e. $r^{SOCS3} = \frac{\mathrm{SOCS3\ (0.17nM)}}{\mathrm{SOCS3\ (0.08nM)}}$ was calculated for the mean of three independent replicates. The resulting ratio was used to normalize SOCS3 protein expression detected on separated blots for cells stimulated with 0.08 nM and 0.17 nM cytokine and thus allows comparison of the results from different experiments.

S3.2 qRT-PCR

Data on SOCS3 mRNA expression in HepG2 or HepG2-IL-6Rα cells obtained by qRT-PCR were normalized to the respective expression in response to 60 min Hy-IL-6 in the presences of CHX (HepG2) or 60 min IL-6 in the presence of CHX (HepG2-IL-6Rα), respectively. Expression at t = 0 min was regarded as background and was thus subtracted from the values for all time points. Mean value and standard deviation of the resulting values over all biological replicates were calculated.

SOCS3 mRNA expression induced in response to different cytokine stimuli, was monitored by separated experiments. For the sake of comparability HepG2 and HepG2-IL-6Rα cells were pretreated with CHX and stimulated with Hy-IL-6 (HepG2) or IL-6 (HepG2-IL-6Rα) (0.08 nM and 0.17 nM) for 60 min, respectively (n = 3 each). The ratio of SOCS3 mRNA expression induced by 0.17 nM and 0.08 nM Hy-IL-6 or IL-6, i.e. $r^{SOCS3mRNA} = \frac{\mathrm{SOCS3\ mRNA\ (0.17nM)}}{\mathrm{SOCS3\ mRNA\ (0.08nM)}}$ was calculated for the mean of three independent experiments. The resulting ratio was used to normalize SOCS3

mRNA expression detected in separated experiments for cells stimulated with 0.08 nM and 0.17 nM cytokine and thus allows comparison of the results.

S4. Supplementary Figures

Figure S1

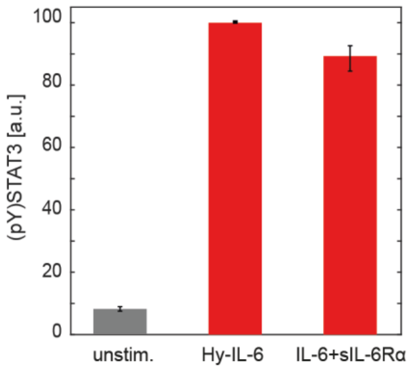

Hy-IL-6 simulates IL-6-induced trans-signalling.

HepG2 cells were stimulated with 0.17 nM Hy-IL-6 or a mixture of 0.17 nM IL-6 and 100 nM sIL-6Rα for 30 min or left untreated. STAT3 phosphorylation was evaluated by intracellular flow cytometry using specific fluorescent antibodies against STAT3 (p)Y705. For independent experiments mean fluorescence of 10^4 cells was calculated and mean fluorescence was normalized to 100 %. Data are given as mean ± STD from n = 3 independent experiments.

Figure S2

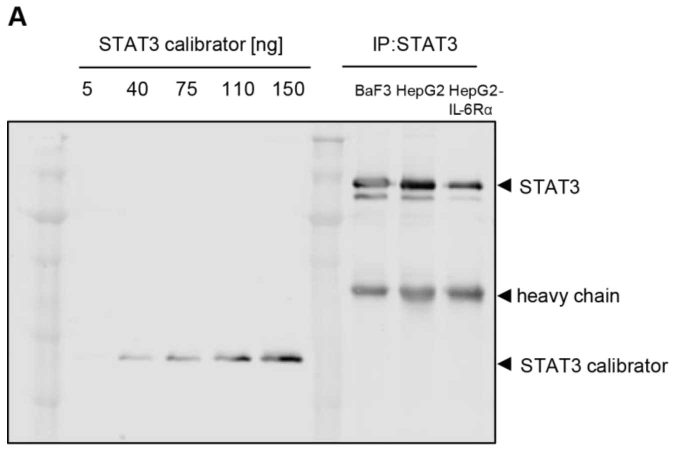

Quantification of STAT3 and (p)STAT3.

(A) STAT3 and phosphorylated STAT3 were isolated from cellular lysates by immunoprecipitation (IP) with antibodies specific for STAT3 and phosphorylated STAT3. Precipitated proteins and a known amount of a recombinant STAT3 calibrator protein were subjected to SDS PAGE and Western Blotting. Proteins were detected with anti-STAT3 antibodies, specific for an epitope present in STAT3, (p)STAT3 and the recombinant STAT3 calibrator protein. **(B)** To correct for incomplete precipitation the initial ratio of STAT3 and (p)STAT3 was determined in aliquots of the cellular lysates before and after precipitation, efficiency of protein precipitation was calculated and considered for the calculation of protein concentrations.

In detail, STAT3 protein was precipitated from lysates of 10^6 unstimulated Ba/F3-gp130-IL-6Rα, HepG2 or HepG2-IL-6Rα cells. (p)STAT3 was precipitated from lysates of 10^6 Ba/F3-gp130-IL-6Rα, HepG2 or HepG2-IL-6Rα cells stimulated with IL-6 or Hy-IL-6 (0.42 nM) for 30 min, respectively. For immunoprecipitation, lysates were incubated with 1 µg of IP antibody at 4°C overnight. The following IP antibodies were used: STAT3 (#9139),

(p)STAT3-Y705 (#9145) (Cell Signalling). Immunocomplexes were isolated using protein G dynabeads (Thermo Fisher Scientific) according to manufacturer‘s instruction. Precipitated proteins and GST-tagged STAT3 calibrator proteins (GST-STAT3 (aa 670-770), Abnova, Taipei, Taiwan) were subjected to SDS-PAGE, and Western blotting was performed. Antigens were detected by incubation with anti-STAT3 antibody (1:1,000) (#9139) (Cell Signaling) followed by incubation with IR Dye-coupled secondary antibodies (1:10^4) (LI-COR, Lincoln, NE, USA). Near infrared fluorescence was detected on an Odyssey Classic Imager (Li-COR). Quantification of bands was performed using Image Studio 4.0 (Licor). The efficiency of immunoprecipitation was controlled by determining STAT3 and (p)STAT3 amount in the lysate before and after immunoprecipitation. 15 µl aliquots of the lysates were taken before and after IP. Subsequently, samples were analysed by SDS-PAGE and Western blotting. Antigens were detected by incubation with specific antibodies against STAT3, (p)STAT3, and HSC70 as described above. Relative amounts of STAT3 and (p)STAT3 respectively were normalized with respect to HSC70 expression. The corresponding values were used to calculate residual STAT3 and (p)STAT3 after precipitation and thereby efficiency of precipitation. Absolute amounts of STAT3 and (p)STAT3 were corrected by the rate of efficacy of precipitation. The cell volume of HepG2 cells was estimated to be 1.6 pl based on the average cell diameter of trypsinized HepG2 cells of 14.5 µm and assuming a spherical shape. The volume of Ba/F3 cells was estimated to be 0.525 pl based on a diameter of 10 µm. Representative quantification of STAT3 from n=7 independent experiments is shown.

Figure S3

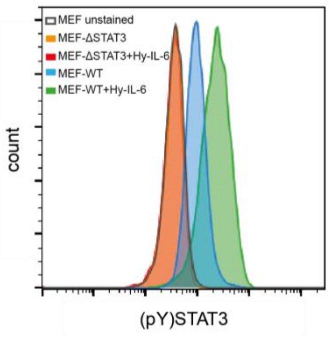

Validation of the specificity of the fluorescent antibody against STAT3 (p)Y705

Specificity of fluorescent antibodies against STAT3 (p)Y705 was validated using STAT3-deficient MEF cells. Immortalised MEF and MEF STAT3$^{-/-}$ cells were grown in DMEM (Thermo Fisher Scientific) supplemented with FCS (10 %), streptomycin and penicillin (each 100 µg/ml] at 37 °C in a water saturated atmosphere containing 5 % CO_2. A total of 10^6 cells were cultured on a 6 cm dish for 24 h. Prior to stimulation, cells were washed with PBS and subsequently starved in 2 ml medium without FCS and antibiotics for 2 h. Cells were treated with 0.17 nM Hy-IL-6 (Conaris, Kiel, Germany) for 15 min. After stimulation cells were detached from cell culture dishes with 1 ml Accutase (Biowest, Nuaillé, France, Cat. No. L0950-100). For fixation 100 µl of the cell suspension was mixed with 100 µl paraformaldehyde and incubated for 10 min at 37 °C followed by centrifugation (230 g at 4 °C for 5 min). Cells were suspended in ice cold 90 % methanol and incubated on ice for 10 min. Subsequently, cells were washed twice with cold BSA-EDTA-Buffer (2 % BSA, 2 mM EDTA in PBS) and incubated with fluorophore-coupled antibody (1:200) against (p)STAT3 (#557814) (BD Biosciences, Franklin Lakes, NJ, USA) for 30 min. Cells were washed two times in BSA-EDTA Buffer and applied to FACS analysis (FACS Canto II (BD Biosciences)). Data were evaluated using FlowJo (Treestar, Ashland, OR, USA). Representative results of n=3 independent experiments are shown.

Figure S4

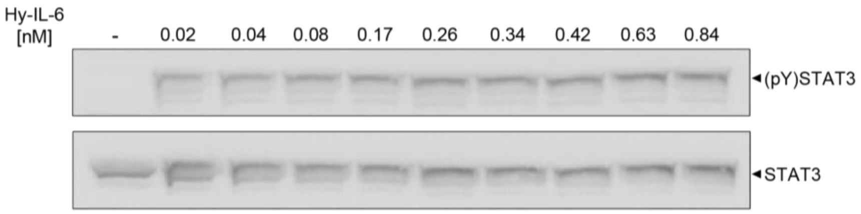

Dose-dependent Hy-IL-6-induced STAT3 phosphorylation using Western Blotting.

HepG2 cells were stimulated with indicated amounts of Hy-IL-6 for 30 min. STAT3 phosphorylation and expression of STAT3 were evaluated by Western blotting. Expression of STAT3 served as loading control. Representative results of n = 3 independent experiments are shown.

Figure S5

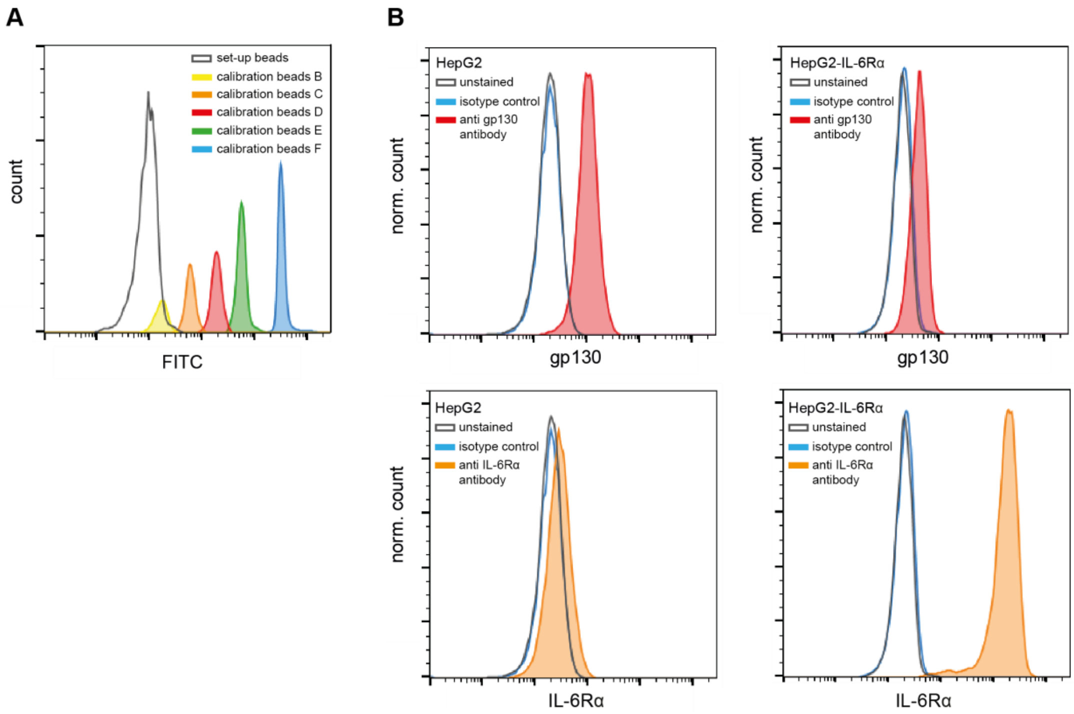

Quantification of gp130 and IL-6Rα cell surface expression.

The amount of gp130 and IL-6Rα on the cell surface was analysed using QIFIKIT, a bead-based FACS assay, according to manufacturer`s instruction (Agilent, Santa Clara, CA, USA). In brief, cells were detached from the cell-culture dish with Accutase and incubated with primary antibodies against gp130 (1:50, BR-3, Hölzel Diagnostika, Cologne, Germany), IL-6Rα (1:100, BR-6, Hölzel Diagnostika) or control IgG (Biolegend, San Diego, CA, USA) for 30 min. Next, cells and calibration beads were stained with FITC-coupled secondary antibody (1:50, Agilent). Cells as well as beads were analysed using FACS Canto II (BD Biosciences). Data were analysed using flowjo (Treestar, Ashland, OR, USA). **(A)** Representative FACS analysis of QIFIKIT calibration beads stained with FITC-coupled secondary antibody. **(B)** Representative FACS analysis of HepG2 and HepG2-IL-6Rα cells stained with anti gp130 or anti IL-6Rα primary antibody and FITC-stained secondary antibody. Representative results of n = 4 independent experiments are shown.

Figure S6

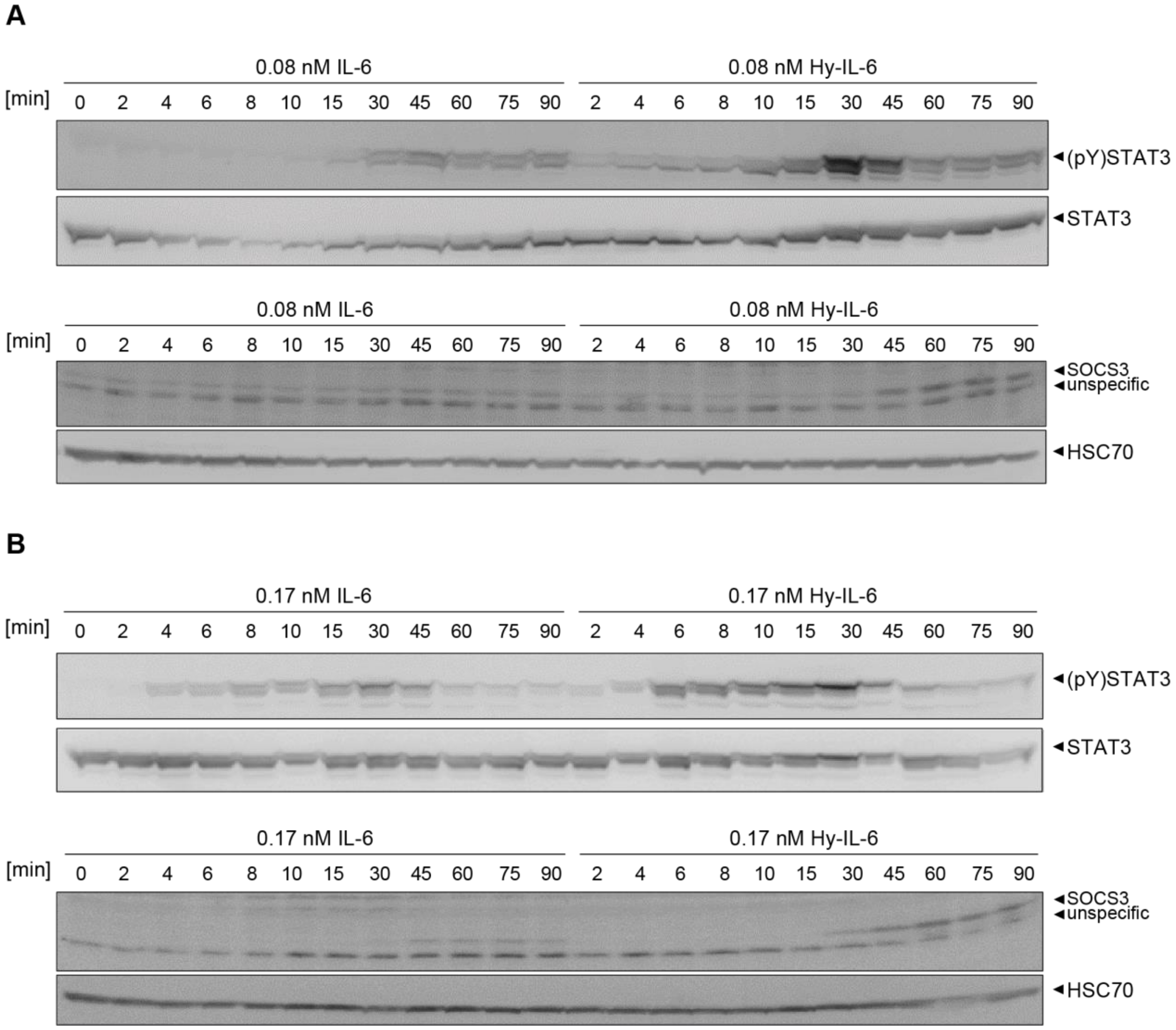

Raw data of Figure 1A and B.

HepG2 cells were stimulated with 0.08 nM **(A)** or 0.17 nM **(B)** IL-6 or Hy-IL-6. STAT3 phosphorylation and expression of STAT3 protein, SOCS3 protein, and HSC70 protein were evaluated by Western blotting. Expression of STAT3 and HSC70 served as loading control. Representative results of n = 3 independent experiments are shown.

Figure S7

A

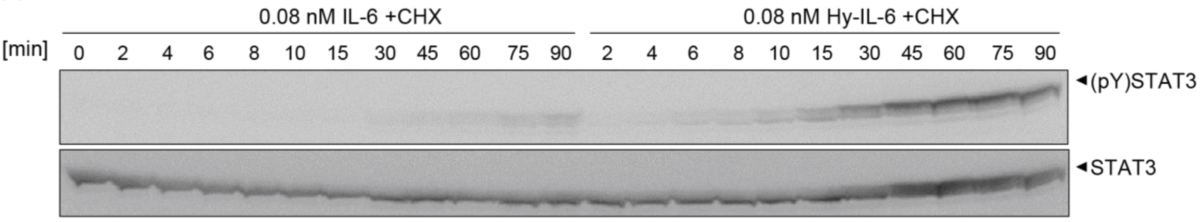

B

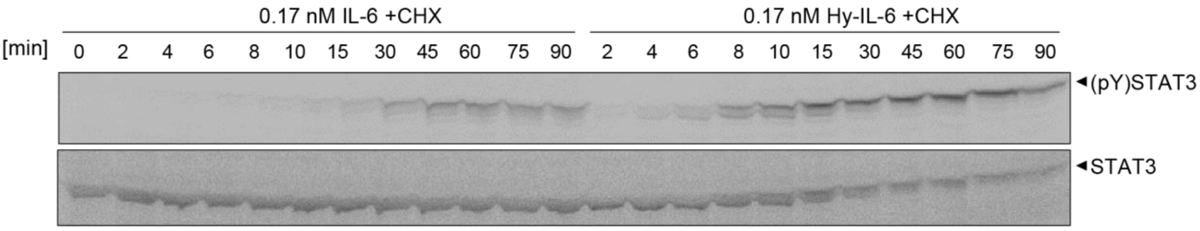

Raw data of Figure 3C and D.

HepG2 cells were pretreated with cycloheximide for 30 min and subsequently stimulated with **(A)** 0.08 nM or **(B)** 0.17 nM IL-6 and Hy-IL-6 as indicated. STAT3 phosphorylation and expression of STAT3 were evaluated by Western blotting. Expression of STAT3 served as loading control. Representative results of n = 3 independent experiments are shown.

Figure S8

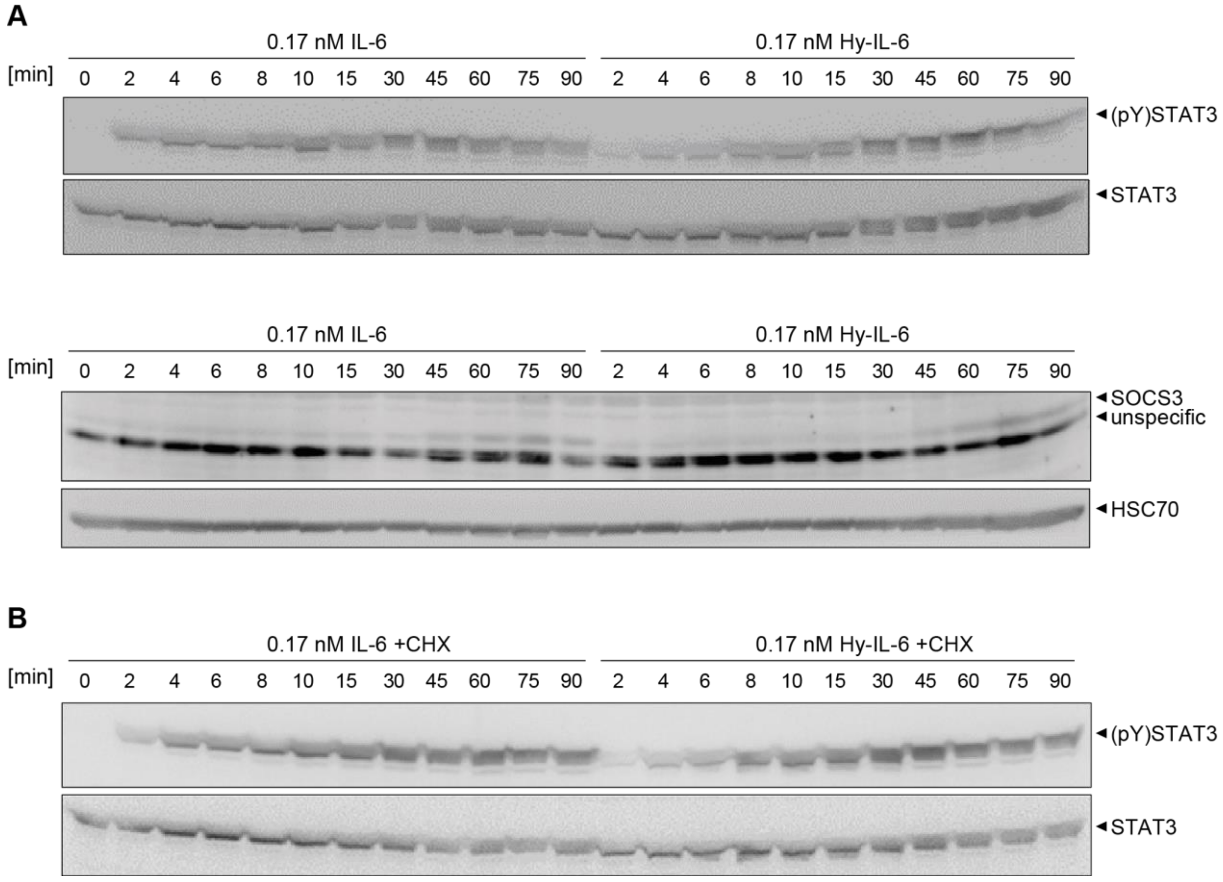

Raw data of Figure 6B and D.

(A) HepG2-IL-6Rα cells were stimulated with IL-6 and Hy-IL-6 (0.17 nM). STAT3 phosphorylation and protein expression of STAT3, SOCS3, and HSC70 were evaluated by Western blotting. Expression of STAT3 and HSC70 served as loading controls. Representative results of n = 3 independent experiments are shown. **(B)** HepG2-IL-6Rα cells were pretreated with cycloheximide for 30 min and subsequently stimulated with IL-6 and Hy-IL-6 (0.17 nM). STAT3 phosphorylation and STAT3 expression were evaluated by Western blotting. STAT3 expression served as loading control. Representative results of n = 3 independent experiments are shown.

Figure S9

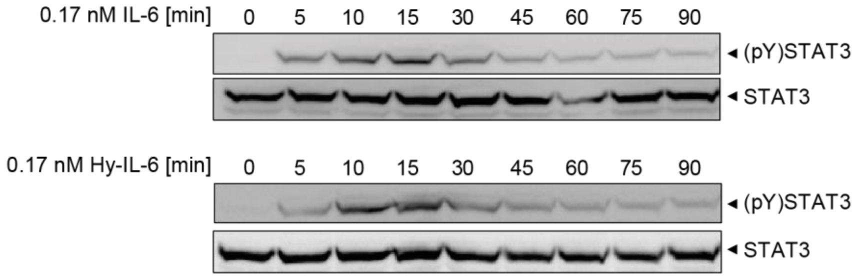

Raw data of Figure 7B.

Ba/F3-gp130-IL-6Rα cells were stimulated with IL-6 and Hy-IL-6 (0.17 nM). STAT3 phosphorylation and STAT3 expression were evaluated by Western blotting. STAT3 expression served as loading control. Representative results of n = 3 independent experiments are shown.

S5. References

1. Borchers S, Rumschinski, P., Bosio, S., Weismantel, R., Findeisen, R.: A set-based framework for coherent model invalidation and parameter estimation of discrete time nonlinear systems**.** In *48th IEEE Conference on Decision and Control (CDC) Shanghai, China*. 2009: 6786-92.
2. Rumschinski P, Borchers S, Bosio S, Weismantel R, Findeisen R: Set-base dynamical parameter estimation and model invalidation for biochemical reaction networks. *BMC Syst Biol* 2010, **4:**69.
3. Streif S, Savchenko A, Rumschinski P, Borchers S, Findeisen R: ADMIT: a toolbox for guaranteed model invalidation, estimation and qualitative-quantitative modeling. *Bioinformatics* 2012, **28:**1290-1.
4. Pietzko D, Zohlnhöfer D, Graeve L, Fleischer D, Stoyan T, Schooltink H, Rose-John S, Heinrich PC: The hepatic interleukin-6 receptor. Studies on its structure and regulation by phorbol 12-myristate 13-acetate-dexamethasone. *J Biol Chem* 1993, **268:**4250-8.
5. Baran P, Hansen S, Wätzig GH, Akbarzadeh M, Lamertz L, Huber HJ, Ahmadian MR, Moll JM, Scheller J: The balance of interleukin (IL)-6, IL-6.soluble IL-6 receptor (sIL-6R), and IL-6.sIL-6R.sgp130 complexes allows simultaneous classic and trans-signaling. *J Biol Chem* 2018, **293:**6762-75.
6. Hibi M, Murakami M, Saito M, Hirano T, Taga T, Kishimoto T: Molecular cloning and expression of an IL-6 signal transducer, gp130. *Cell* 1990, **63:**1149-57.
